# Repression of Mcl-1 expression by the CDC7/CDK9 inhibitor PHA-767491 overcomes bone marrow stroma-mediated drug resistance in AML

**DOI:** 10.1038/s41598-018-33982-y

**Published:** 2018-10-25

**Authors:** Eimear O’ Reilly, Sukhraj Pal S. Dhami, Denis V. Baev, Csaba Ortutay, Anna Halpin-McCormick, Ruth Morrell, Corrado Santocanale, Afshin Samali, John Quinn, Michael E O’Dwyer, Eva Szegezdi

**Affiliations:** 1 Apoptosis Research Centre, School of Natural Sciences, National University of Ireland Galway, Galway, Ireland; 2 Centre for Chromosome Biology, School of Natural Sciences, National University of Ireland Galway, Galway, Ireland; 3 Hematology, Beaumont Hospital, Dublin, Ireland; 4 Apoptosis Research Centre, School of Medicine, National University of Ireland Galway, Galway, Ireland; 5 HiDucator Ltd, Kangasala, Finland

## Abstract

Acute myeloid leukaemia (AML) is an aggressive cancer with 50–75% of patients relapsing even after successful chemotherapy. The role of the bone marrow microenvironment (BMM) in protecting AML cells from chemotherapeutics and causing consequent relapse is increasingly recognised. However the role that the anti-apoptotic Bcl-2 proteins play as effectors of BMM-mediated drug resistance are less understood. Here we show that bone marrow mesenchymal stromal cells (BMSC) provide resistance to AML cells against BH_3_-mimetics, cytarabine and daunorubicin, but this is not mediated by Bcl-2 and/or Bcl-X_L_ as previously thought. Instead, BMSCs induced Mcl-1 expression over Bcl-2 and/or Bcl-X_L_ in AML cells and inhibition of Mcl-1 with a small-molecule inhibitor, A1210477, or repressing its expression with the CDC7/CDK9 dual-inhibitor, PHA-767491 restored sensitivity to BH_3_-mimetics. Furthermore, combined inhibition of Bcl-2/Bcl-X_L_ and Mcl-1 could revert BMSC-mediated resistance against cytarabine + daunorubicin. Importantly, the CD34^+^/CD38^−^ leukemic stem cell-encompassing population was equally sensitive to the combination of PHA-767491 and ABT-737. These results indicate that Bcl-2/Bcl-X_L_ and Mcl-1 act in a redundant fashion as effectors of BMM-mediated AML drug resistance and highlight the potential of Mcl-1-repression to revert BMM-mediated drug resistance in the leukemic stem cell population, thus, prevent disease relapse and ultimately improve patient survival.

## Introduction

Acute myeloid leukemia (AML) is a complex disease driven by a combination of genetic and epigenetic alterations in the hematopoietic stem or progenitor cells. Despite our increasing understanding of the molecular aberrancies that drive AML, up to 20–30% of young and 40–50% of older AML patients are refractory to treatment. Furthermore, the risk of relapse is high, between 50–75% depending on age^1^. The prognosis following relapse is poor and at this stage, no good treatment strategies available^2^. As our understanding of the molecular aberrations driving AML increases, a number of targeted therapeutics, such as protein kinase inhibitors (FLT3, PI3K, Akt, Erk or Pim inhibitors), inhibitors of DNA methylating- and acetylating enzymes, such as DNMT1, DNMT3, DOT1L and HDACs or BH_3_-mimetics against anti-apoptotic Bcl-2 proteins are being developed^3,4^. While the development of these inhibitors is progressing rapidly, understanding the role of the bone marrow microenvironment (BMM) in controlling the epigenetic landscape and driving survival signalling in AML cells is lagging behind. Underlining its importance, bone marrow-mediated protection was found to be the major cause of low FLT3-inhibitor efficacy^5,6^.

The most studied mechanism by which bone marrow stromal cells (BMSCs) induce drug resistance is the activation of pro-survival signal transduction, typically culminating in the upregulation of Bcl-2 (BCL2) and/or Bcl-X_L_ (BCL2L1)^7,8^. Induction of anti-apoptotic Bcl-2 proteins is an inherent feature of normal differentiation of leukocytes as Bcl-2 proteins provide survival advantage to the properly formed mature cells. For example, Mcl-1 (MCL1) is required for the survival of hematopoietic stem cells (HSC)^9^, common myeloid progenitors (CMP) and common lymphoid progenitors (CLP), Bcl-2 is induced during the selection of T and B lymphocytes while Bcl-X_L_ (BCL2L1) is critical for erythrocyte-^10,11^, megakaryocyte-^12^ and platelet survival^13^, and A1 (BCL2A1) supports neutrophil survival^14^.

Increased Bcl-2 expression is also a characteristic of several haematological malignancies, including chronic lymphocytic leukemia (CLL) and AML. The notion that leukemic cells become dependent on anti-apoptotic Bcl-2 protein expression for survival is proven by the potent effect of the Bcl-2/Bcl-X_L_/Bcl-W inhibitor, ABT-737 and its Bcl-2-selective variant, ABT-199^15^. The ability of anti-apoptotic Bcl-2 proteins to drive drug resistance is also well established. Accordingly, ABT-737 and/or ABT-199 have been shown to sensitise isolated AML cells to 5-azacytidine^16^, FLT3 inhibitors^17^ as well as docetaxel^18^.

Here we determined the role of anti-apoptotic Bcl-2 proteins as effectors of bone marrow stroma-mediated drug resistance in AML blasts and the CD34^+^/CD38^−^ cells representing a population enriched for leukemic stem cells (LSC)^19^. We show that bone marrow stromal cells (BMSCs) provide resistance against BH_3_-mimetics, cytarabine (AraC) and daunorubicin (DnR) and that this protection is also pronounced in the CD34^+^/CD38^−^ cell population. We show that inhibition of Bcl-2 and Bcl-X_L_ with ABT-737 is not sufficient to revert BMSC-mediated drug resistance against AraC + DnR. On the other hand, BMSC-mediated drug resistance was associated with increased Mcl-1 expression. Furthermore, Mcl-1 inhibition with A1210477 or repression with PHA-767491 could revert drug resistance mediated by BMSCs. Importantly, repression of Mcl-1 expression with the dual CDC7/CDK9 inhibitor PHA-767491 equally sensitised the CD34^+^/CD38^−^ cell population offering a strategy to eradicate the main cell population responsible for disease relapse.

## Results

### Bone marrow mesenchymal stromal cells protect AML cells from therapeutic drugs

**Figure 1:**
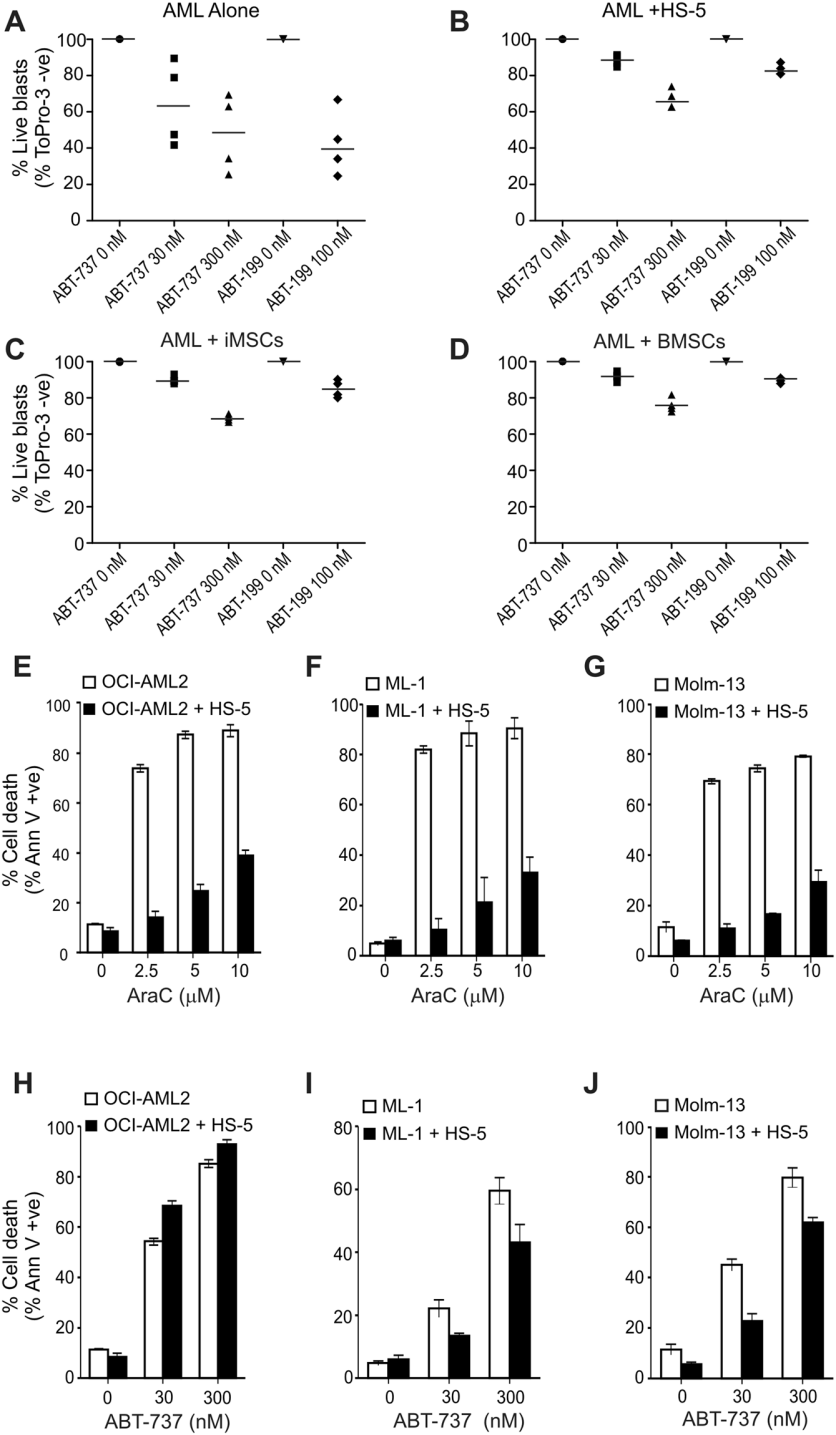
Bone marrow mesenchymal cells reduce AML sensitivity to ABT- 737 and cytarabine. (**A**–**D**) Immortalised, healthy-donor derived BMSCs can replicate the protective effect of the patients’ own BMSCs against BH_3_-mimetics. Bone marrow-derived mononuclear cells from AML patients were culture alone (**A**) or over 3 different bone marrow stromal cell layers; HS-5 cells (**B**), iMSCs (**C**) and the patients’ own BMSCs (**D**) for 24 h after which the cells were treated with ABT-737 or ABT-199 for another 24 h. Induction of cell death in the AML population was determined with ToPro-3 staining using flow cytometry. The graphs show % live blasts from 4 different patients normalised to the untreated control. (**E**–**J**) HS-5 BMSCs provide protection against cytarabine (AraC) and ABT-737 in AML cell lines. OCI-AML2, ML-1 and Molm-13 cells were cultured in direct contact with HS-5 BMSCs for 24 h followed by treatment with a dosage of AraC (**E**–**G**) or ABT-737 (**H**–**J**) for another 24 h and induction of cell death was quantified by flow cytometry using Annexin V. The graphs show the average percentage of dead cells ± stdev from at least three independent repeats.

In order to determine the effect of anti-apoptotic Bcl-2 proteins in drug resistance mediated by the BMM, a layered stroma-AML co-culture system has been set up. AML cell lines or primary AML blasts were cultured on a monolayer of BMSCs in direct contact. As a model of BMSCs, HS-5 cells, an immortalised healthy donor-derived BMSC cell line, were used. HS-5 cells were chosen over primary BMSCs of AML patients, as the latter were found to be prone to senescence under *ex vivo* culture^20^. As HS-5 cells are only a clone of immortalised BMSCs, they may not represent the full spectrum of function that primary BMSCs have. Thus we tested how faithfully they could replicate the effect of patients’ own BMSCs. To this end, primary AML cells were cultured alone, on the patients’ own BMSCs or on two different immortalised BMSC lines; the commercially available HS-5 cells and a non-commercially available hTERT immortalised BMSC cell line that we named iMSC. After 24 h, the cultures were treated with ABT-737 or ABT-199 and induction of cell death was quantified. Both HS-5 cells and iMSCs could both replicate the effect of the patients’ own BMSCs in providing a comparable level of resistance to AML cells against both drugs (Fig. 1A–D, Suppl. Fig. 1).

Cytokine expression as a measure of their ability to model features of the BMM has also been determined using a cytokine proteome array (Bio-Techne, Suppl. Table 1). HS-5 cells secreted the cytokines and chemokines characteristic of the bone marrow^21^, including granulocyte-colony stimulating factor (G-CSF), angiopoietin 1 (ANGPT1), ANGPT2, growth differentiation factor 15 (GDF15), osteopontin, interleukin 1β (IL-1β), fibroblast growth factor 2 (FGF2), C-C motif chemokine ligand 5 (CCL5), CCL7, C-X-C motif chemokine ligand 10 (CXCL10), CXCL12 (SDF1α) (for the complete list of secreted cyto/chemokines, see Suppl. Table 1).

The broader effect of BMSCs on AML drug sensitivity was next tested using AraC, DnR and ABT-737 in both AML cell lines and primary AML cells. OCI-AML2, ML-1 and Molm-13 cells were co-cultured with HS-5 cells for 24 h followed by treatment with a dosage of AraC or ABT-737 for 24 h and cell viability was determined with Annexin V staining. With the exception of ABT-737 treatment of OCI-AML2 cells, all co-cultures showed a significantly reduced sensitivity (Fig. 1E–J).

**Figure 2:**
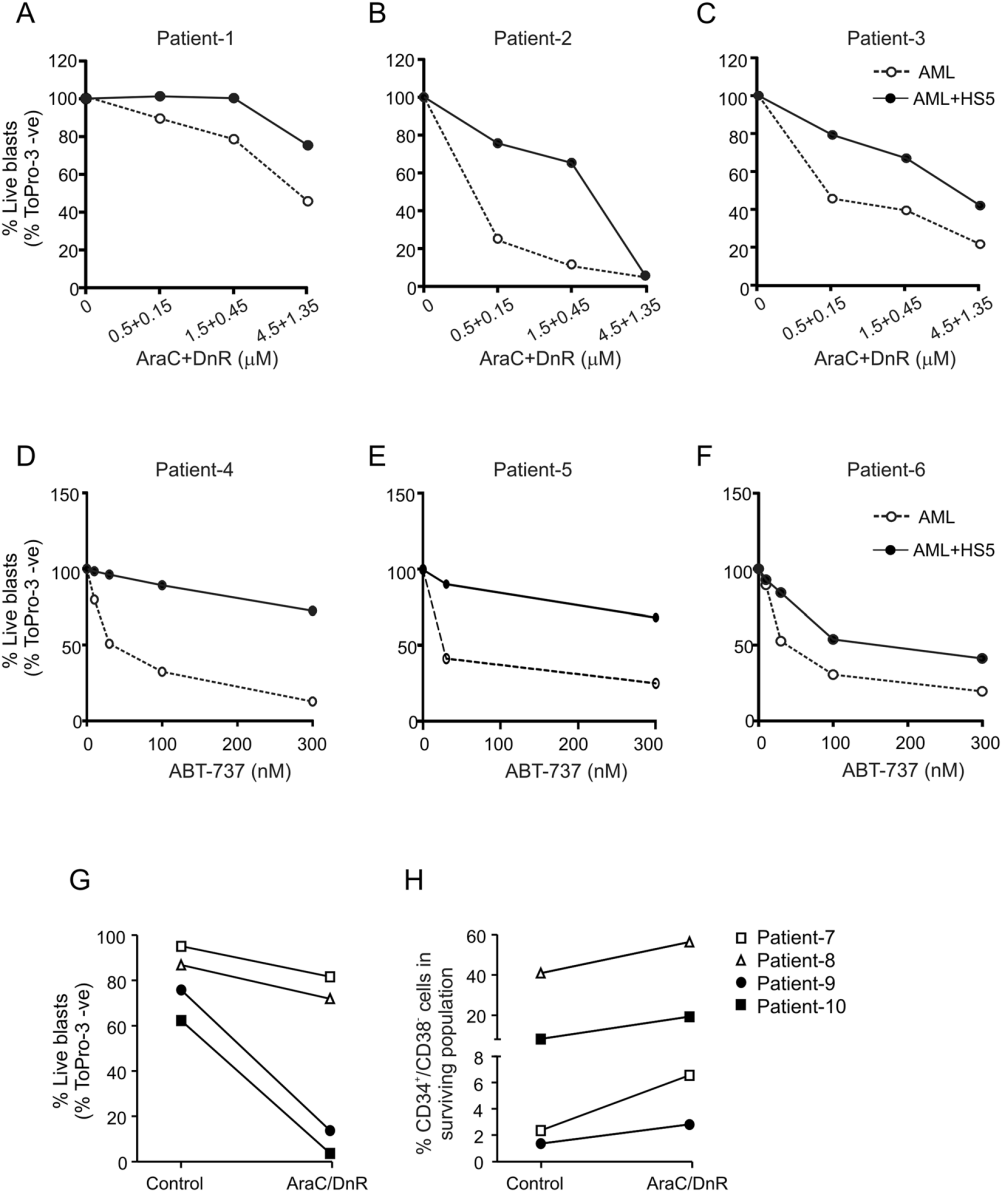
Primary AML blasts gain resistance against cytotoxic therapeutics when cultured in contact with BMSCs. Bone marrow-derived mononuclear cells from AML patients were cultured either alone or with HS-5 BMSCs for 24 h followed by exposure to a 3:1 molar ratio of AraC and DnR (**A**–**C**) or to a dosage of ABT-737 (**D**–**F**) for 24 h. Induction of cell death in the AML blasts was determined with ToPro-3 staining using flow cytometry. The graphs show percentage live blasts normalised to the untreated control. (**G** and **H**) HS-5 BMSCs provide resistance to the LSC-encompassing CD34^+^/CD38^−^ population against AraC + DnR. BM-derived AML blasts from 4 patients were cultured with HS-5 BMSCs and treated with AraC + DnR as in sections (**A**–**C**). The graph shows the percentage of CD34^+^/CD38^−^ cells within the surviving cell fraction as a measure of their relative enrichment.

Similar effects were found using AML blasts. The mononuclear cell fraction isolated from bone marrow aspirates of AML patients (AML blasts) were cultured on HS-5 cells for 24 h followed by exposure to a 3:1 molar ratio of AraC and DnR (corresponding to the molar ratio of the two drugs used in the clinic (7 + 3 therapy^22^) or a dosage of ABT-737 for 24 h. Induction of cell death in the AML blasts was determined using the viability dye, ToPro-3. Similar to the cell lines, AML blasts gained resistance against both AraC + DnR and ABT-737 when cultured with BMSCs (Fig. 2A–F). Of note, the resistance against DnR + AraC provided by HS-5 BMSCs was also pronounced in the CD34^+^/CD38^−^ population shown by their enrichment in the surviving cell fraction (Fig. 2G,H).

### AML cells residing in the bone marrow microenvironment have increased Mcl-1 expression

**Figure 3:**
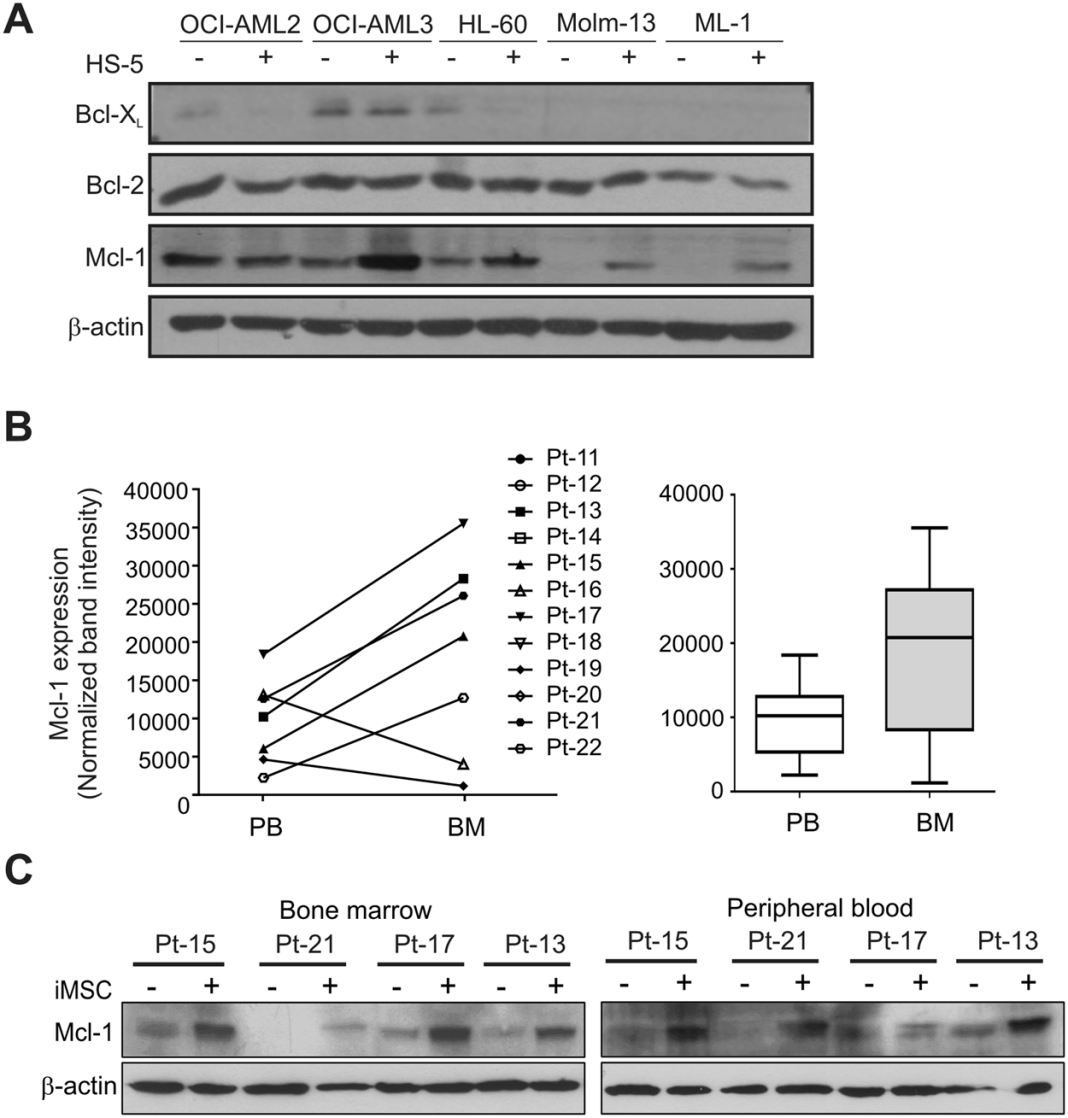
Bone marrow stromal cells induce Mcl-1 expression in AML cells. (**A**) Expression of anti-apoptotic Bcl-2 family proteins in AML cells cultured on HS-5 BMSCs. OCI-AML2, OCI-AML3, HL60, ML-1, Molm-13 cells were cultured alone or in direct contact with HS-5 cells for 24 h and changes in the expression of Bcl-2, Bcl-X_L_ and Mcl-1 were determined with Western blotting. Levels of β-actin were measured as loading control. (**B**) Expression of Mcl-1 protein in the bone marrow- and peripheral blood-derived AML blasts. Whole cell lysates of mononuclear cells isolated from paired bone marrow aspirates and peripheral blood from 12 AML patients were analysed for the expression of Mcl-1 by Western blotting and quantified with densitometry. The left hand graph shows the expression values of individual patients and the right hand graph shows the value distribution as a box-plot. The p-value was determined using a paired t-test. (**C**) Mcl-1 expression in AML blasts cultured alone or with BMSCs. Mcl-1 protein expression was detected in bone marrow- and peripheral blood-derived MNCs from 4 AML patient cultured alone or on iMSC BMSCs for 24 h. Levels of β-actin were measured as a loading control.

To determine the contribution of anti-apoptotic Bcl-2 proteins to BMM-driven drug resistance, we determined the effect of HS-5 BMSCs on the expression of anti-apoptotic Bcl-2 proteins. A panel of AML cell lines (OCI-AML2, OCI-AML3, HL60, ML-1, Molm-13) were cultured with HS-5 cells for 24 h and changes in the expression of Bcl-2, Bcl-X_L_ and Mcl-1 were determined with Western blotting. Interestingly, while HS-5 BMSCs did not induce significant change in the expression of Bcl-2 and Bcl-X_L_, in 4 out of the 5 cell lines tested, Mcl-1 induction was detected (Fig. 3A).

To gain insight whether the BMM exerts the same effect *in vivo*, we compared the mRNA expression of Bcl-2, Bcl-X_L_ and Mcl-1 between bone marrow (BM)-residing and peripheral blood (PB)-circulating AML blasts. To this end, mRNA expression data from the Gene Expression Omnibus dataset, GDS3057 was extracted^23^. The housekeeping genes, GPI (Glucose-6-Phosphate Isomerase), PSMB2 (Proteasome Subunit Beta 2) and EMC (ER Membrane Protein Complex Subunit) were used for normalisation. Bcl-2 and Bcl-X_L_ expression was comparable between BM and PB. Mcl-1 expression on the other hand showed the opposite trend, it appeared to be lower in PB than in BM (Suppl. Fig. 2A), however, it did now each a level of significance.

Because there are no gene expression datasets with matched BM and PB samples (i.e. BM and PB sample were not from the same patient) and because mRNA expression may not mirror the actual protein expression, we have tested Mcl-1 protein expression in paired BM and PB samples of 12 AML patient samples. Mcl-1 was expressed in 7 out of the 12 samples, out of which 5 have shown higher expression of Mcl-1 in the BM in comparison to the PB (Fig. 3B and Suppl. Fig. 2B). Statistical testing showed no significant difference in Mcl-1 expression between BM and PB with all samples included. After excluding the samples that showed no detectable Mcl-1 expression, a significant difference was apparent between Mcl-1 expression in BM versus PB (p = 0.033). We then tested whether AML blasts also show Mcl-1 induction upon contact with BMSCs, similar to AML cell lines. For this, BM-derived versus PB-derived AML blasts were cultured in contact with BMSCs (iMSC) for 24 h and Mcl-1 protein expression was determined with Western blotting. Contact with iMSCs induced Mcl-1 expression all 4 samples tested (Fig. 3C, Suppl. Fig. 2C, D). Taken together, these experiments show that in a subset, but not in all patients, the BMM drives Mcl-1 expression in the residing AML blasts.

**Figure 4:**
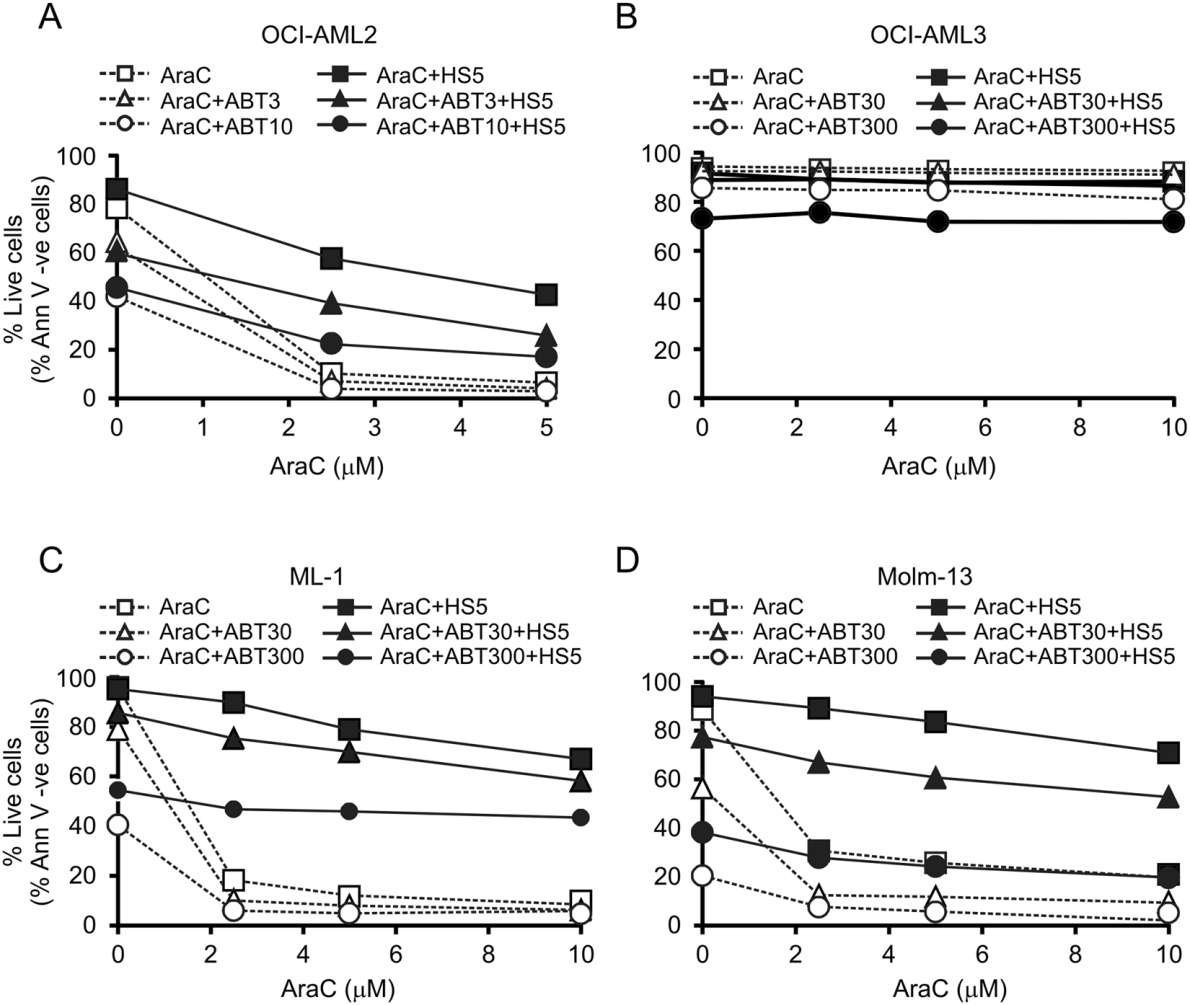
Inhibition of Bcl-2 and Bcl-X_L_ fails to revert BMSC-driven drug resistance. OCI-AML2 (**A**), OCI-AML3 (**B**), ML-1 (**C**) and Molm-13 (**D**) cells were cultured on a HS-5 BMSC layer for 24 h before exposing the cells to a combination of AraC and ABT-737 at the doses indicated (ABT3 = 3 nM, ABT10 = 10 nM, ABT30 = 30 nM and ABT300 = 300 nM) for an additional 24 h. The graphs show the average percentage of live cells ± stdev quantified with Annexin V staining from three independent experiments.

To assess the contribution of anti-apoptotic Bcl-2 proteins to BMM-mediated AML drug resistance, we tested if inhibition of Bcl-2/Bcl-X_L_ can revert BMSC-mediated resistance to chemotherapeutics. To this end, AML cell lines were cultured with HS-5 BMSCs for 24 h before exposing the cells to AraC for an additional 24 h in the presence or absence of ABT-737. The doses of ABT-737 for each cell line has been selected based on their sensitivity profile (Suppl. Fig. 3). All cell lines, except OCI-AML3 (Fig. 4B), showed sensitivity to ABT-737 administered as a single agent, indicating that AML cells depend on Bcl-2/Bcl-X_L_ expression for survival even in the BM environment. At the same time, ABT-737 could not revert BMSC-mediated resistance to AraC and DnR, indicating that Bcl-2 and Bcl-X_L_ are not the sole effectors of BMSC-mediated drug resistance (Fig. 4).

### Combined ABT-737 and PHA-767491 treatment targets both the bulk AML blasts and the CD34^+^/CD38^−^ cell population

Given that expression of Mcl-1 consistently increased in AML cells following co-culture with BMSCs as well as reports showing that elevated levels of Mcl-1 are associated with leukemia relapse^24^, we tested whether Mcl-1 contributes to reduced ABT-737 sensitivity mediated by HS-5 BMSCs. As the first approach, we have used the CDC7/CDK9 inhibitor PHA-767491 as inhibition of CDK9 represses transcription leading to reduced expression of short half-life time proteins, including Mcl-1^25,26^.

**Figure 5:**
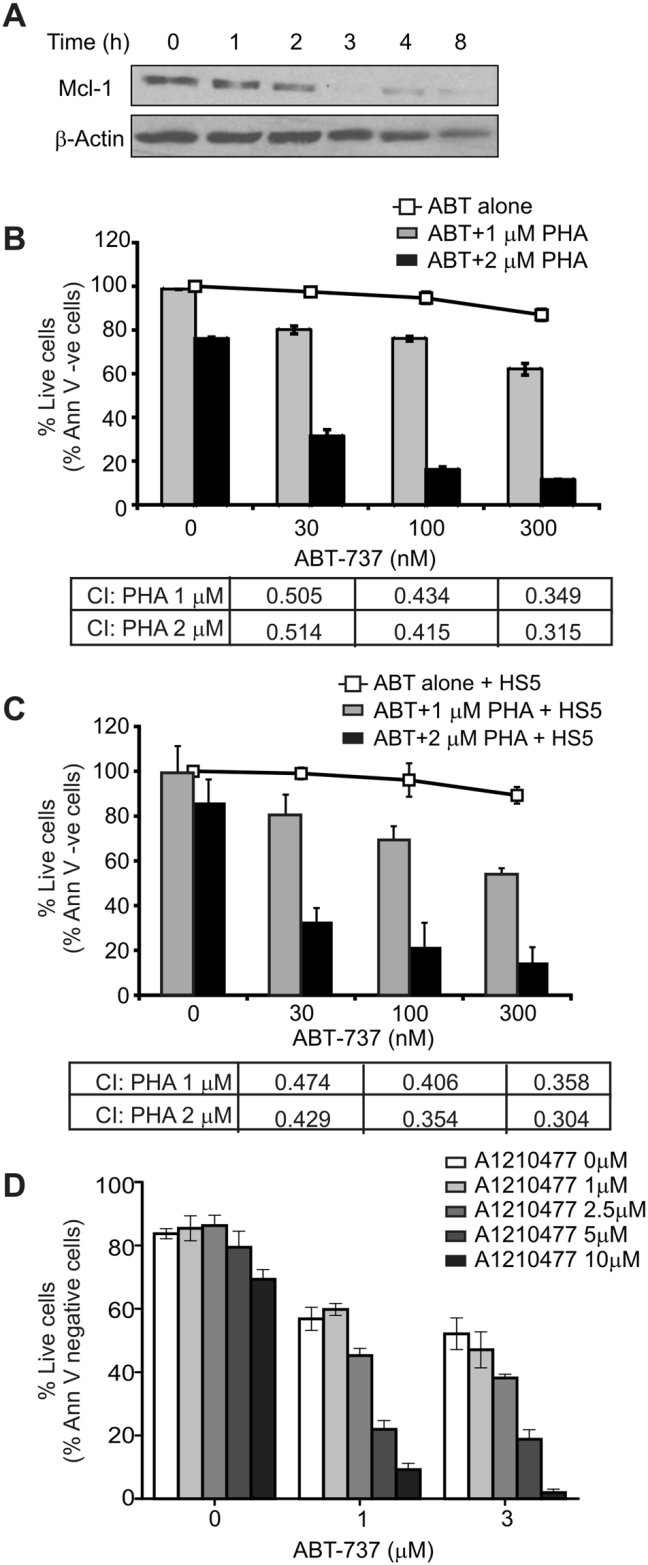
AML cells supported by BMSCs display high sensitivity to ABT-737 upon repression of Mcl-1 expression. (**A**) Repression of Mcl-1 expression in response to treatment with the dual CDC7/CDK9 inhibitor PHA-767491. OCI-AML3 cells were treated with 2 µM PHA-767491 and whole cell lysates were harvested over an 8 h time course and Mcl-1 expression was monitored by Western blotting. Expression of β-actin was detected to normalise for protein loading. (**B** and **C**) OCI-AML3 cells were cultured alone (**B**) or over a HS-5 feeder layer (**C**) and were treated with doses of PHA-767491 for 4 h followed by a dosage of ABT-737 for an additional 20 h. Cell death was quantified using Annexin V staining and flow cytometry. The table under the graphs show the calculated combination index (CI) for the PHA + ABT-treated samples. (**D**) OCI-AML3 cells were treated with the Mcl-1 inhibitor, A1210477, at the concentrations indicated for 4 h followed by treatment with ABT-737 for a further 20 h. Cell death was quantified using Annexin V and flow cytometry. The graphs show average percentage live cells ± stdev from three independent experiments.

The ABT-737-resistant OCI-AML3 cells (characterised by high Mcl-1 expression) were cultured alone or on a HS-5 BMSC-layer for 24 h. The cultures were pre-treated with PHA-767491 for 4 h followed by a dosage of ABT-737 for an additional 20 h. Repression of Mcl-1 expression was monitored by Western blotting (Fig. 5A) and induction of cell death was quantified with Annexin V staining. Treatment with 2 µM of PHA-767491 alone triggered a modest induction of apoptosis, but, it potently sensitised the cells to ABT-737 (Fig. 5B,C). Importantly, the drug combination retained full efficacy in the presence of HS-5 BMSCs (Fig. 5C). At this concentration, PHA-767491 downregulated Mcl-1 expression after 3 h of exposure onward (Fig. 5A). Using the Chou-Talalay median effect equation, the combination index (CI) was calculated (table under the graphs in Fig. 5B,C). At each drug concentration, the CI index was well below 1.0, indicating a potent synergy between ABT-737 and PHA-767491.

To confirm that the mechanism of PHA-767491-mediated sensitization involved Mcl-1, a small molecule Mcl-1 inhibitor, A1210477, was employed. A1210477 is a selective, small-molecule inhibitor of Mcl-1 developed by the Soeurs lab^27^ shown to have a high selectivity to Mcl-1 over other Bcl-2 family members, multiple kinases and G-protein coupled receptors. Similarly to treatment with PHA-767491, A1201477 was able to sensitise OCI-AML3 cells to ABT-737 (Fig. 5D).

**Figure 6:**
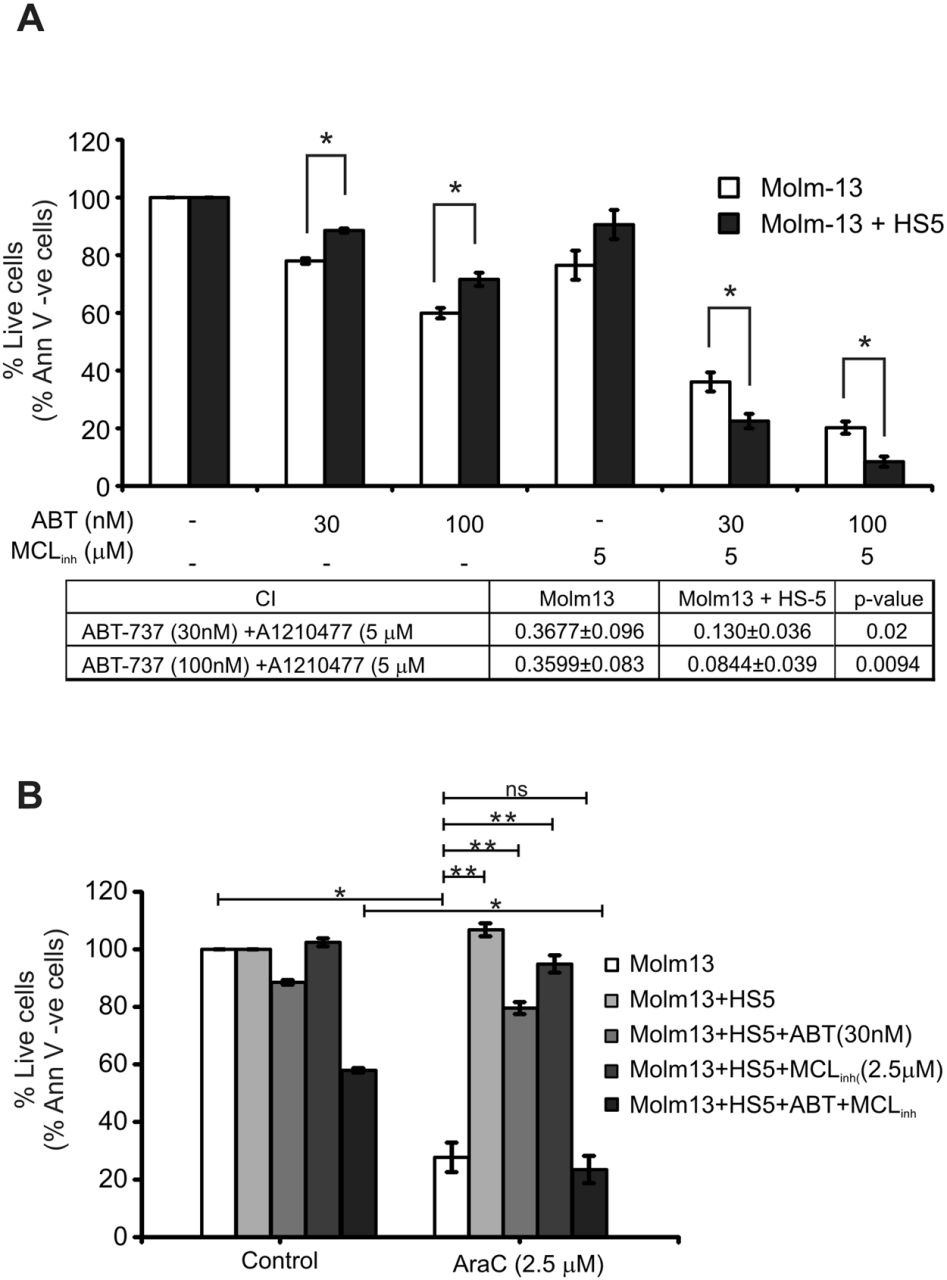
Mcl-1 is an effector of bone marrow stroma-driven drug resistance. (**A**) Effect of Mcl-1 inhibition on BMSC-mediated resistance against ABT-737. Molm-13 cells were cultured alone, or in contact with HS-5 BMSCs for 24 h followed by treatment with ABT-737 (ABT) and the small molecule Mcl-1 inhibitor, A1210477 (MCL_inh_) for 24 h. For the combination treatment, Molm-13 cells were pre-treated with MCL_inh_ for 4 h, followed by treatment with ABT for 24 h. Cell viability was determined with Annexin V staining. The table under the graphs shows the CI values of the combination treatment. *indicates significant difference determined with student t-test (p < 0.05). (**B**) Bcl-2 and Mcl-1 act in a redundant manner as effectors of BMSC-driven resistance against cytarabine (AraC). Molm-13 cells cultured as described above were treated with AraC (2.5 µM) in the presence of ABT-737 (ABT, 30 nM), A1210477 (MCL_inh_, 2.5 µM, 4 h pre-treatment) or both for 24 h and Molm-13 cell death was quantified with Annexin V staining. The graphs show the average percentage of live cells ± stdev from three independent experiments (stars indicate significant differences with *meaning p < 0.05 and **meaning p < 0.005, determined with student t-test).

In order to corroborate the above result, the role of Mcl-1 in BMSC-mediated drug resistance was tested in a second AML cell line, with lower Mcl-1 expression. As opposed to OCI-AML3 cells, Molm-13 cells have a low baseline Mcl-1 expression but, they show Mcl-1 induction upon contact with BMSCs (Fig. 3A) and increased resistance to ABT-737 and AraC (Fig. 1G,J). Molm-13 cells were cultured alone or in contact with HS-5 BMSCs as before and exposed to ABT-737, with or without a 4 h pre-treatment with A1210477, for 20 h and induction of cell death was determined with Annexin V staining (Fig. 6A). Co-culture with BMSCs induced a moderate, but consistent protection against both ABT-737 and A1210477. At the same time, A1210477 pre-treatment sensitised Molm-13 cells to ABT-737 both in single culture and in co-culture with HS-5 cells (Fig. 6A, CI values are under the graph). Notably, the effect of the Mcl-1 inhibitor was more pronounced under the co-culture conditions, reflected also by lower CI indices, indicating that Mcl-1 is an effector of BMSC-mediated ABT-737 resistance (Fig. 6A).

Similar results were found when the effect of Mcl-1 inhibition on BMSC-mediated AraC-resistance was tested. While ABT-737 could not revert BMSC-mediated AraC resistance, additional inhibition of Mcl-1 fully restored AraC-sensitivity (Fig. 6B). Of note, inhibition of Mcl-1 in the absence of Bcl-2 inhibition could not revert AraC-sensitivity either, indicating a redundant function between Bcl-2 and Mcl-1 in BMSC-mediated drug resistance.

**Figure 7:**
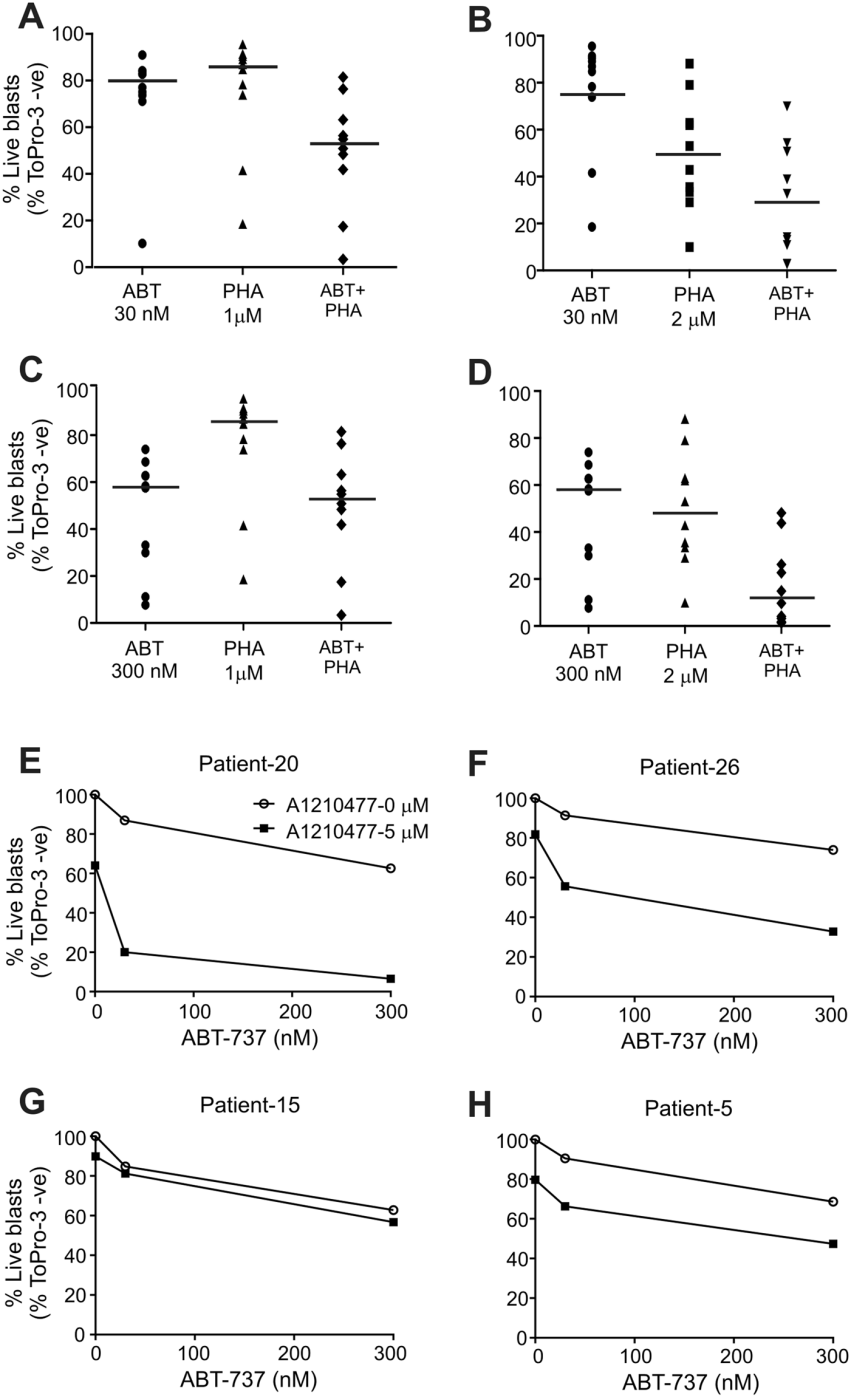
Repression of Mcl-1 expression with PHA-767491 or pharmacological inhibition with A1210477 reverts BMSC-driven drug resistance of AML blasts. (**A**–**D**) Repression of Mcl-1 with PHA-767491 enhances sensitivity to ABT-737. AML blasts from 10 patients were cultured on an HS5-BMSC monolayer for 24 h and then treated with ABT-737 with or without a 4 h pre-treatment with the CDC7/CDK9 inhibitor, PHA-767491 (30 nM ABT + 1 μΜ PHA (**A**) 30 nM ABT +2 μΜ PHA (**B**) 300 nM ABT +1 μΜ PHA (**C**) 300 nM ABT +2 μΜ PHA (**D**)). Induction of cell death was quantified with ToPro-3 viability staining in the bulk AML population. Graphs (**A**–**D**) show the percentage of live blasts as a dot plot with the line indicating the median response. (**E**–**H**) AML blasts from 4 patients were co-cultured with HS-5 BMSCs for 24 h followed by treatment with ABT-737 (30 and 300 nM) alone or in combination and with the Mcl-1 inhibitor, A1210477 (5 µM) for 24 h. Induction of cell death was measured with ToPro-3 staining for the bulk AML population. The graphs show the percentage of live blasts normalised to the untreated control.

The finding that AML cells have enhanced sensitivity to the combined inhibition of Bcl-2/Bcl-X_L_ and Mcl-1 when they are in contact with BMSCs was further explored using AML blasts (Fig. 7). Samples from 10 patients were cultured on a HS-5 BMSC monolayer for 24 h, followed by treatment with ABT-737 for 24 h (30 nM and 300 nM) with or without a 4 h pre-treatment with PHA-767491 (1–4 µM). Except for one patient (Patient 2), whose cells displayed a high sensitivity for ABT-737, the combination of ABT-737 and PHA-767491 showed a synergistic effect (Fig. 7A–D, Suppl. Fig. 4, CI indices in Suppl. Table 2). We also carried out these experiments using the patients’ own BMSCs and found a similar synergistic effect (Suppl. Fig. 5A, C, E and G), corroborating the findings. Because the LSC population often has higher drug resistance driving relapse, the sensitivity of the CD34^+^/CD38^−^ LSC-encompassing population was also determined (Suppl.Fig. 4). Looking at the effect of the two drugs individually, the CD34^+^/CD38^−^ population showed higher resistance to ABT-737 in 3 out of the 10 samples (Patient 24, 25 and 6) and in 2 samples, they were more resistant to PHA-767491 (Patient 24 and 5). Importantly, the combination treatments showed that PHA-767491 could sensitise the ABT-737 resistant cells and the drug combination had a potent cytotoxic effect. The clinical data (Suppl. Table 3) of the tested samples shows that patients with refractory AML are also sensitive to the combination of ABT-737 and PHA-767491, highlighting a potential patient cohort that could benefit from this drug combination.

Because PHA-767491 is likely to repress the expression of a number of genes, not only Mcl-1, Mcl-1 was also inhibited using A1210577 to prove that sensitisation to ABT-737 required Mcl-1 repression. 4 patient samples were treated as before, only replacing PHA-767491 with A1210577 (5 μM, Fig. 7E–H). As a single agent, A1210477 had a much lower cytotoxic effect than PHA-767491 in line with its expected, lower non-specific (Mcl-1 independent) effects. A1210477 sensitised 2 out of the 4 samples to ABT-737 (patient 20 and 26), confirming that repression of Mcl-1 is a key mechanism through which PHA-767491 sensitises AML cells under BMSC support to ABT-737. The combination of ABT-737 with A1210477 was also tested using matched BMSCs. Inhibition of Mcl-1 was equally efficient in sensitizing the AML blast cultured with their matched BMSCs, giving an indication for similar potency *in vivo* (Suppl. Fig. 5B, D, F and H). To confirm that repression or inhibition of Mcl-1 sensitizes AML blasts under stromal support, a knockdown of Mcl-1 was carried out on primary AML blasts. AML blasts from 2 patients, whose blasts showed resistance to ABT-737 were transfected with siRNA against Mcl-1 using nucleofection (Suppl. Fig. 6A) and co-cultured with iMSCs for 24 h. Cells were then treated with ABT-737 (30 and 300 nM) for 24 h. While we noticed that the transfection itself stressed the cells and reduced Mcl-1 expression, knockdown of Mcl-1 enhanced sensitivity to ABT-737 (Suppl. Fig. 6B).

As ABT-737 is only a proof-of-concept drug, we also tested the clinically relevant variant, ABT-199 (Bcl-2 selective inhibitor). In the 4 patient samples tested, pre-treatment with both PHA-767491 or A1210477 enhanced sensitivity to ABT-199. Of note, the sensitisation appears to be lower than to ABT-737, indication that Bcl-X_L_ may also contribute to drug resistance (Suppl. Fig. 7).

**Figure 8:**
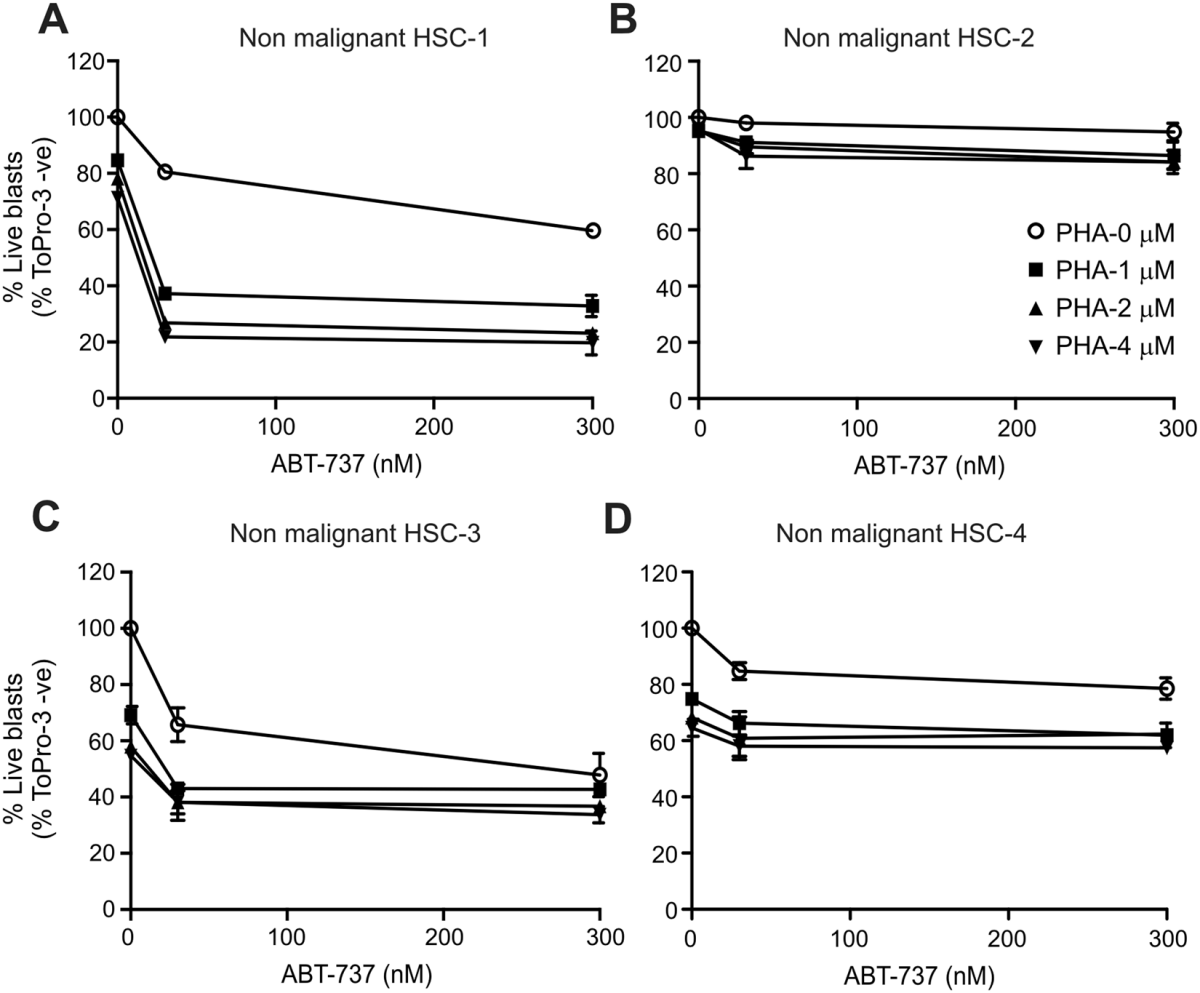
PHA-767491 does not sensitise non-AML HSCs to ABT-737. CD34^+^ non-malignant HSCs were isolated from four patients with non-AML disorders. These cells were cultured on an HS5-BMSC monolayer for 24 h and then treated with ABT-737 (30 and 300 nM) with or without a 4 h pre-treatment with the CDC7/CDK9 inhibitor, PHA-767491 (1–4 µM). Induction of cell death was quantified with To-Pro-3 staining and flow cytometry. The graphs show the percentage of live blasts. The effect of PHA-767491 alone is shown by the points on the Y axis (zero ABT-737 concentration) and the effect of ABT-737 alone is shown by the PHA 0 µM line (open circles).

Since Bcl-2 proteins, including Mcl-1, play fundamental roles in hematopoietic lineage cells, the potential toxic effect of the combined treatment on normal HSCs was measured. For this purpose, we obtained CD34^+^ HSCs from patients with a disease not associated with aberrant HSCs (the disease profile of these samples is listed in Suppl. Table 3, patient 27–30). The samples were analysed in the same co-culture settings as before. Non-malignant HSC-1 and -3 showed a partial sensitivity to high dose PHA767491 (Fig. 8A,C) and non-malignant HSC-2 and -4 showed a partial sensitivity to ABT-737 (Fig. 8B,D). However, the drug combination failed to induce a synergistic effect, except in non-malignant HSC-1. Of note, both HSC-1 and HSC-3 samples were from multiple myeloma patients, and the disease background might have had an impact on the behaviour of the HSCs. Overall, these results indicate a possible therapeutic window for the targeting of LSCs with the ABT-737 and PHA-767491 drug combination.

## Discussion

Hematopoietic lineage cells are dependent on the expression anti-apoptotic Bcl-2 proteins for survival. Individual Bcl-2 family members are upregulated at different stages of hematopoietic differentiation as well as by cytokine signals, which allows the survival of the properly formed or antigen-reactive cells.

Malignantly transformed leukemic cells appear to have a tighter dependency on anti-apoptotic Bcl-2 proteins than normal leukocytes. The reason for this is that oncogenic stress and other cellular stresses associated with malignant transformation lead to the induction of pro-apoptotic BH_3_-only proteins, such as Bim in lymphoma development, and unless these are neutralised by anti-apoptotic family members the transformed leukemic cells may not survive^28^. In line with this notion, BH_3_-mimetic drugs, such as the mainstream Bcl-2/Bcl-X_L_/Bcl-W inhibitors ABT-737 and ABT-263 and their Bcl-2-selective derivative, ABT-199 showed remarkable efficacy in clinical trials^29^.

High anti-apoptotic Bcl-2 protein expression does not only promote survival of leukemic cells, but it is also associated with treatment-resistance. High percentage of Bcl-2 positive AML cells correlate with low complete remission rates after intensive chemotherapy^30^ and high Bcl-2 expression levels in AML cells were found to drive resistance against cytarabine^31^. Bcl-X_L_ expression has also been associated with chemoresistance, for example against 5-azacytidine in AML or the topoisomerase inhibitor, etoposide, in B cell leukemia^16,32^.

The effect of the bone marrow microenvironment on drug resistance is well established. However, its effect on the expression of anti-apoptotic Bcl-2 proteins and the impact of BMM-mediated anti-apoptotic Bcl-2 protein expression on resistance against classical chemotherapeutics or BH_3_-mimetics is much less understood. Equally, the effect of the BMM on anti-apoptotic Bcl-2 protein expression in the most resistant LSC population and drug resistance conveyed by them is also poorly investigated.

Here we show that BMSCs drive resistance against the mainstream chemotherapeutics AraC + DnR with the CD34^+^/CD38^−^ cells, representative of an LSC-enriched population, displaying higher resistance than the AML blasts. We found that inhibition of Bcl-2/Bcl-X_L_/Bcl-W with ABT-737 could not reverse this resistance. We also found that the BMM induced Mcl-1 expression both *in vitro* in AML cell lines and *in vivo* in a subset of primary samples, while BMM-mediated Bcl-2 and Bcl-X_L_ induction was less consistent. In line with these findings, Garrido and colleagues have also reported inconsistent or low level induction of Bcl-2 in primary patient AML cells in *ex vivo* BMSC co-cultures^33^.

Normal HSCs are known to depend on Mcl-1 expression for long-term survival. Furthermore, Mcl-1 gene deficiency results in loss of mature B and T lymphocytes over time, demonstrating a central role for Mcl-1 in the long-term maintenance of the mature immune system^34^. Based on these results, Mcl-1 inhibitors were predicted to be toxic. However, while ABT-199 potently kills primary AML blasts, a trend of a negative correlation between Mcl-1 expression and the efficacy of ABT-199 exists^15^. Also, induction of Mcl-1 is frequently observed in relapsed AML^35^ and Mcl-1 induction is recognised as the main mechanism of resistance against ABT-263 and ABT-199^36,37^.

Our finding that the bone marrow stroma drives selective upregulation of Mcl-1 identifies a potential mechanism how ABT-263 and ABT-199 resistance may develop *in vivo*. In line with our findings, the study of Glaser and colleagues showed higher dependency of AML cells on Mcl-1 over healthy HSCs, giving grounds for a possible therapeutic window for Mcl-1 targeting^24^. Recent studies also support the potential of Mcl-1-targeting to overcome drug resistance in AML as well as in other leukaemia types^38^, with selective Mcl-1 inhibitors showing promise in pre-clinical studies^39^.

Mcl-1 has a number of distinguishing features that separates it from Bcl-2 and Bcl-X_L_. It lacks a conserved BH4 domain and it has a very short half-life time due to a P-E-S-T motif in its N-terminal portion (resulting a half-life time of only 2–4 h in most cells)^40,41^. This, combined with the multiple pathways that control its (1) transcription (such as the transcription factors ATF5, E2F1, STAT3, PU.1 and NF-kB), (2) regulate its translation (3) as well as protein stability, create a very dynamic control of Mcl-1 protein levels in response to a wide variety of cellular stresses and signals^41,42^.

To date, there was little focus on the role of Mcl-1 in BMM-driven AML drug resistance. Here we found that Bcl-2 and Mcl-1 are both effectors of bone marrow stroma-mediated drug resistance and they act in a redundant manner, i.e. the presence of either Bcl-2 or Mcl-1 was sufficient to provide resistance since simultaneous inhibition of both proteins was required to restore drug-sensitivity.

The CDC7/CDK9 inhibitor, PHA-767491 represses Mcl-1 expression^43^. CDK9 forms the catalytic core of the positive transcription elongation factor b (P-TEFb). P-TEFb phosphorylates RNA polymerase II and thus initiates the elongation phase of transcription^44^. Inhibition of CDK9 thus leads to the depletion of proteins with short half-life times, such as Mcl-1^25^. Previous studies have shown that combining CDK9 inhibitors with Bcl-2 inhibitors, such as ABT-737 and ABT-199, can overcome drug resistance in a synergistic manner^45–47^. We found that PHA-767491-mediated downregulation of Mcl-1 was very potent in sensitising both AML cell lines and primary AML blasts to ABT-737 and to the recently FDA-approved ABT-199. It has to be noted, that ABT-199 appeared to be inferior in inducing AML cell death compared to ABT-737 in our studies, indicating a possible role of Bcl-X_L_ as well.

As shown by Jilg and colleagues, the CD34^+^ population showed sensitivity to ABT-737^48^. Our studies confirm these findings, but also show that in some patients the CD34^+^/CD38^−^ population is resistant to ABT-737. Importantly, PHA-767491 restored the sensitivity of resistant CD34^+^/CD38^−^ cells to ABT-737. These results highlight the role of Mcl-1 in addition to Bcl-2 as a key effector of BMM-mediated pro-survival signalling and consequent drug resistance. At the same time, non-malignant CD34^+^ HSCs were not sensitised to ABT-737 by PHA-767491, indicating that Mcl-1 has a differential role in CD34^+^ cells of AML versus non-malignant HSCs thus offering a therapeutic window to target LSCs. Chemoresistant AML may be a potential patient cohort who could benefit from treatment with ABT-737 and PHA-767491 or other CDK9 inhibitors, as samples from refractory AML patients showed equally high sensitivity to the ABT-737 + PHA-767491 treatment. In this regard, a recent report highlighted that feedback activation of STAT3, a key transcriptional regulator of Mcl-1 was induced by the BMM and drove chemotherapy-resistance^49^.

Mcl-1 has been shown to play a central role in emergency hematopoiesis, i.e. the regeneration of hematopoietic lineage cells after stress. It is likely that chemotherapy triggers the same effect^50^. This emphasizes the need to consider pharmaceutical inhibition of Mcl-1 to target LSCs and reduce the occurrence of relapse. However, the potential toxic effect of Mcl-1 inhibitors cannot be neglected.

Therapies that target pathways that upregulate Mcl-1 expression in neoplastic cells as opposed to direct inhibition of Mcl-1 may be less toxic. In this regard, CDK9 has been shown to be aberrantly activated by oncogenic fusion proteins (such as MLL-fusion proteins) driving lymphoid and myeloid leukemia^51–53^. NF-κB (p65) also requires P-TEFb for transcription elongation^54^. As NF-κB signalling is often elevated in AML and it can drive Mcl-1 expression, this represents another mechanism how inhibition of CDK9 can repress abnormal Mcl-1 expression^54^. Finally the Gandhi laboratory reported, although in CLL not AML, that stroma-driven resistance to apoptosis of CLL cells was associated with a cascade of transcriptional events that included increased phosphorylation of RNA Pol II on serine residues at positions 2 and 5 (the phosphorylation site of CDK9), leading to increased rate of global RNA synthesis, and amplification of Mcl-1 transcript levels^55^. These reports provide a strong rationale for targeting Mcl-1 gene expression as opposed to direct inhibition of the Mcl-1 protein (like a BH_3_-mimetic)

In conclusion, the results presented here indicate that Bcl-2/Bcl-X_L_ and Mcl-1 act in a redundant fashion as effectors of BMM-mediated AML drug resistance and indicate that for BH3-mimetic-based treatment of AML the focus must be broadened from sole targeting of Bcl-2 to the additional inhibition or repression of Mcl-1.

## Materials and Methods

### Reagents

All reagents were purchased from Sigma, unless stated otherwise. ABT-737 and A1210477 from Selleck Chemicals, ABT-199 from Active Biochem and PHA-767491 (provided by Corrado Santocanale, NUI Galway) were dissolved in dimethyl sulfoxide (DMSO). AraC and DnR (Sigma) were dissolved in water. Carboxyfluorescein succinimidyl ester (CFSE) (Biolegend) was dissolved in DMSO. Annexin V was purchased from Immunotools. ToPro-3 was purchased from Molecular Probes.

### Cell culture

OCI-AML2, OCI-AML3, HL-60, ML-1 and Molm-13 cell lines were cultured in RPMI-1640 medium (Gibco) containing 10% heat-inactivated HyClone fetal bovine serum (HI-FBS, ThermoFisher), penicillin (100 U/ml), streptomycin (100 μg /ml) and 2 mg/ml L-glutamine. HS-5 cells were cultured in DMEM supplemented with 10% HI-FBS, penicillin (100 U/ml), streptomycin (100 μg/ml). iMSCs (hTERT immortalized primary BMSCs from a healthy donor) and primary BMSCs were cultured in alpha-MEM (Sigma) containing 10% HI-FBS, penicillin (100 U/ml) and streptomycin (100 μg /ml). To enable identification of BMSCs in analyses, HS-5 cells were transfected with GFP, while iMSCs and primary BMSCs were stained with the green fluorescent cell-tracker CFSE by incubating the 1 × 10^6^ cells/ml with 5 μM CFSE for 30 minutes prior to seeding.

Primary AML samples were generated from bone marrow aspirates by isolating the mononuclear cell (MNC) fraction with Ficoll gradient-centrifugation as described before^56^. MNCs were stored in liquid nitrogen until use. Upon revival of the cells, viability was determined with trypan blue staining. Only samples with viability above 60% were used. Primary AML blasts were grown in αMEM (Sigma) containing 10% HI-FBS, penicillin (100 U/ml), streptomycin (100 μg /ml), L-glutamine (2 mg/ml) and sodium pyruvate (1 mM).

### Ethics Statement

Ethical approval was obtained from the Research Ethics Committee of University College Hospital, Galway and NUI Galway. The ethical approval confirmed that all methods were carried out according to the regulation of NUI Galway and all applied protocols have been approved by the research ethics committees. Informed consent was obtained from all participants.

### Immunophenotyping and viability assay

MNCs were incubated with fluorochrome-conjugated anti-CD34 ((phycoerythrin, PE) and anti-CD38 (allophycocyanine (APC)-H7) antibodies (BD Bioscience, San Diego, USA) in PBS/1% BSA (Sigma) for 30 minutes on ice in the dark. After washing off unbound antibodies, cells were stained with ToPro-3 (Molecular Probes) according to the manufacturers’ protocol. The samples were analysed using BD FACS Canto II flow cytometer (BD Bioscience, San Diego, USA) by collecting 100,000 events AML blast gate. HS-5 cells were excluded by gating out GFP^+^/FSС^high^ events. The detailed gating strategy is shown in Suppl. Fig. 8.

In the AML cell lines cell death was quantified with Annexin V-FITC or propidium iodide (PI) staining. Cells were collected and stained with Annexin V-FITC in Annexin V buffer (10 mM HEPES/NaOH, pH 7.5, 140 mM NaCl, 2.5 mM CaCl_2_) or propidium iodide (1 μg/ml) for 15 min on ice in the dark. Samples were analyzed on a FACS Canto II flow cytometer. Statistical analysis was performed using FCSExpress (DeNovo Software inc, USA) and GraphPad Prism (GraphPad Software inc., La Jolla, USA) software packages.

### Western blotting

Cells were lysed in whole cell lysis buffer (1% Igepal-630, 20 mM HEPES pH 7.5, 350 mM NaCl, 1 mM MgCl_2_, 0.5 mM EDTA, 0.1 mM EGTA, 0.5 mM DTT, and protease inhibitor cocktail). Proteins (30 µg) were electrophoresed and transferred onto nitrocellulose membrane (Protran). After blocking the blots were incubated with rabbit polyclonal antibodies against Mcl-1 Cell Signaling Technologies (CST), actin (Sigma) and mouse monoclonal antibodies against Bcl-X_L_ (Santa Cruz) and Bcl-2 (Santa Cruz). For detection, horseradish peroxidase-conjugated goat secondary antibodies were used. Protein bands were visualized with SuperSignal® West Pico Chemiluminescent Substrate (Pierce) or Immobilon western HRP substrate (Millipore) on X-ray film (Agfa). Quantification of the Western blots by densitometry was conducted using Image Studio Lite (Li-Cor). Statistical analysis was performed on normalised band intensities using paired student t-test.

### Gene expression analysis

Gene expression profiles were investigated in an open-access dataset of 64 samples (Gene Expression Omnibus: GDS3057)^57^. GDS3057 contained 26 AML samples (7 bone marrow (BM) and 19 peripheral blood (PB)) and 38 healthy donors (18 BM and 20 PB). The BM and PB samples were not matched (not from the same patients). Shapiro test was used to see normality of data groups. Two-tailed t-test was used to estimate significance of difference of mean gene expressions using the R statistical environment.

### siRNA transfection

Primary AML mononuclear cells (1 × 10^6^) were pelleted and resuspended in 100 µl of Nucleofector solution T (Amaxa, Lonza) containing 50 nM siRNA against Mcl-1 (5′-GUGUUAAGAGAAGCA CUAA-3′) or GFP (as a non-specific, control siRNA, 5′-GGCUACGUCCAGGAGCGCAC C-3′, Ambion). Cells were transfected by nucleofection using the U-15 program, as per manufacturer’s protocol (Amaxa)^58^.

### Statistical Analysis

Drug combination index (CI) was calculated using the Chou-Talalay median effect equation using the CompuSyn software package. CI values below 1 (CI < 1) indicated potent synergy. Statistical analyses applied for specific methods are detailed under each corresponding method subheading.

## Electronic supplementary material

Supplementary Information

## Data Availability

All data generated or analysed during this study is included in this published article (and its Supplementary Information files).
