# Supplementary material for: Repression of Mcl-1 expression by the CDC7/CDK9 inhibitor PHA-767491 overcomes bone marrow stroma-mediated drug resistance in AML
Source: Sci Rep. 2018 Oct 25;8:15752. doi: 10.1038/s41598-018-33982-y (PMC6202320; doi:10.1038/s41598-018-33982-y)
Supplement: Supplementary file 1 — Supplementary Information [file 41598_2018_33982_MOESM1_ESM.pdf]

Supplementary Information

Repression of Mcl-1 expression by the CDC7/CDK9 inhibitor PHA-767491 overcomes bone marrow stroma-mediated drug resistance in AML

Eimear O' Reilly*, Sukhraj Pal S. Dhami*, Denis V. Baev, Csaba Ortutay, Anna Halpin-McCormick, Ruth Morrell, Corrado Santocanale, Afshin Samali, John Quinn, ME O'Dwyer, Eva Szegezdi

Supplementary Figure 1

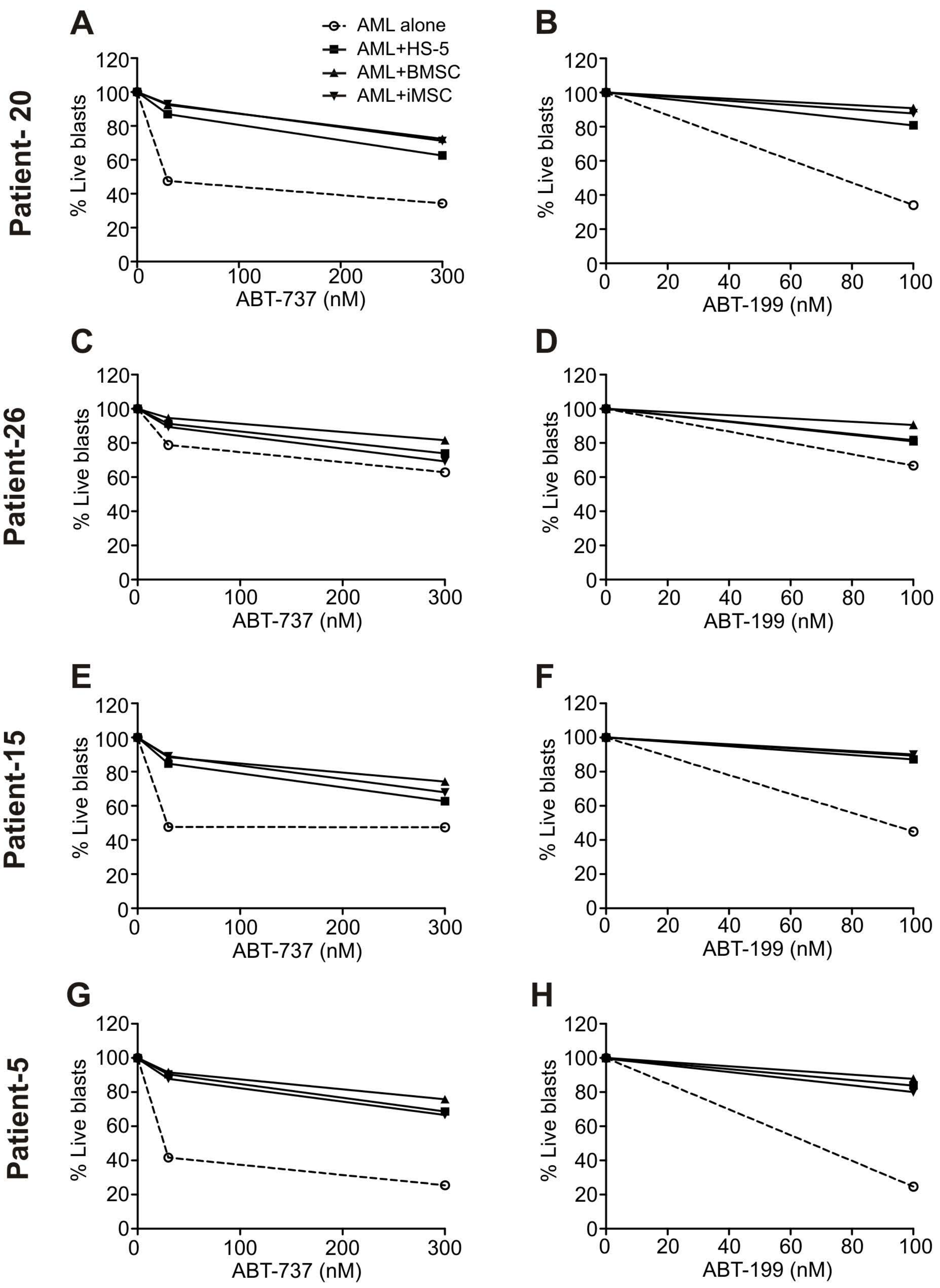

Supplementary Figure 1. HS-5 cells can model bone marrow-mediated AML drug resistance. Primary AML cells from 4 patients were cultured for 24 hours alone (A), on matched primary bone marrow mesenchymal stromal cells (BMSC, B), iMSCs (C) or HS-5 cells (D) for 24 h and treated with the indicated doses of ABT-737 (A, C, E, G) or ABT-199 (B, D, F, H). Induction of cell death was measured with ToPro-3 viability staining and flow cytometry.

Supplementary Figure 2

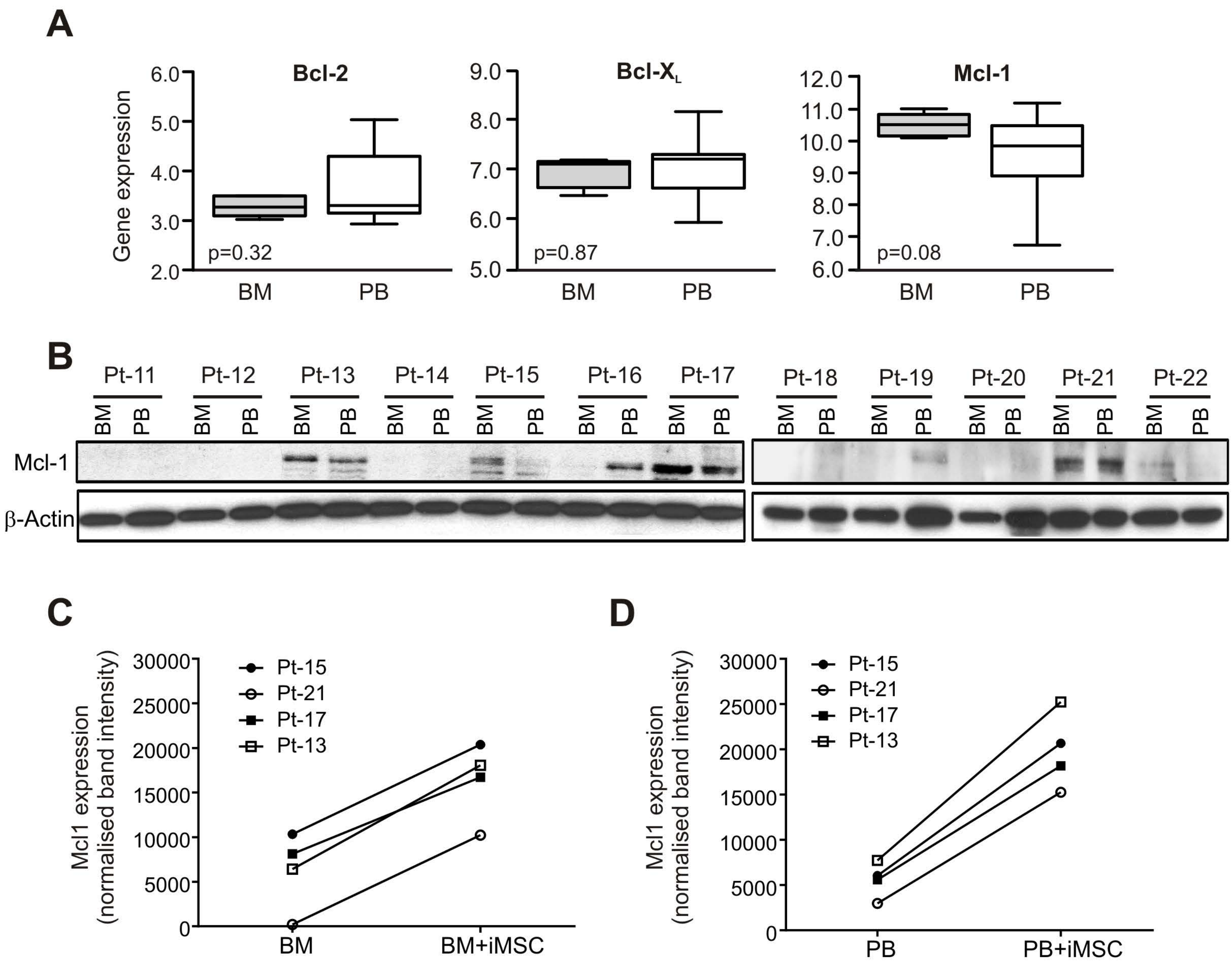

Supplementary Figure 2. Mcl-1 protein expression in bone marrow- and peripheral blood-residing AML blasts. (A) Box plot representation of Bc-2, Bcl-XL and Mcl-1 mRNA expression in AML blasts isolated from either bone marrow (BM) or peripheral blood (PB). Note: the plotted expression data was extracted from the Gene Expression Omnibus dataset (GDS3057) of non-matched BM and PB samples. (B) Mcl-1 protein expression in matched BM- and PB-derived AML blasts. Whole cell lysates from matched BM- and PB-derived AML blasts were analysed for Mcl-1 expression using Western blotting. β-actin expression is shown as loading control. (C and D) BMSCs drive Mcl-1 expression in co-residing AML blasts. The graphs show densitometric quantitation of Mcl-1 expression in AML blasts isolated from BM (C) or PB (D) cultured alone or on BMSCs (iMSCs) for 24 h. Mcl-1 expression determined from whole cell lysates with Western blotting normalised to β-actin is shown as a dot plot. The original Western blot is shown in Fig. 3D.

Supplementary Figure 3

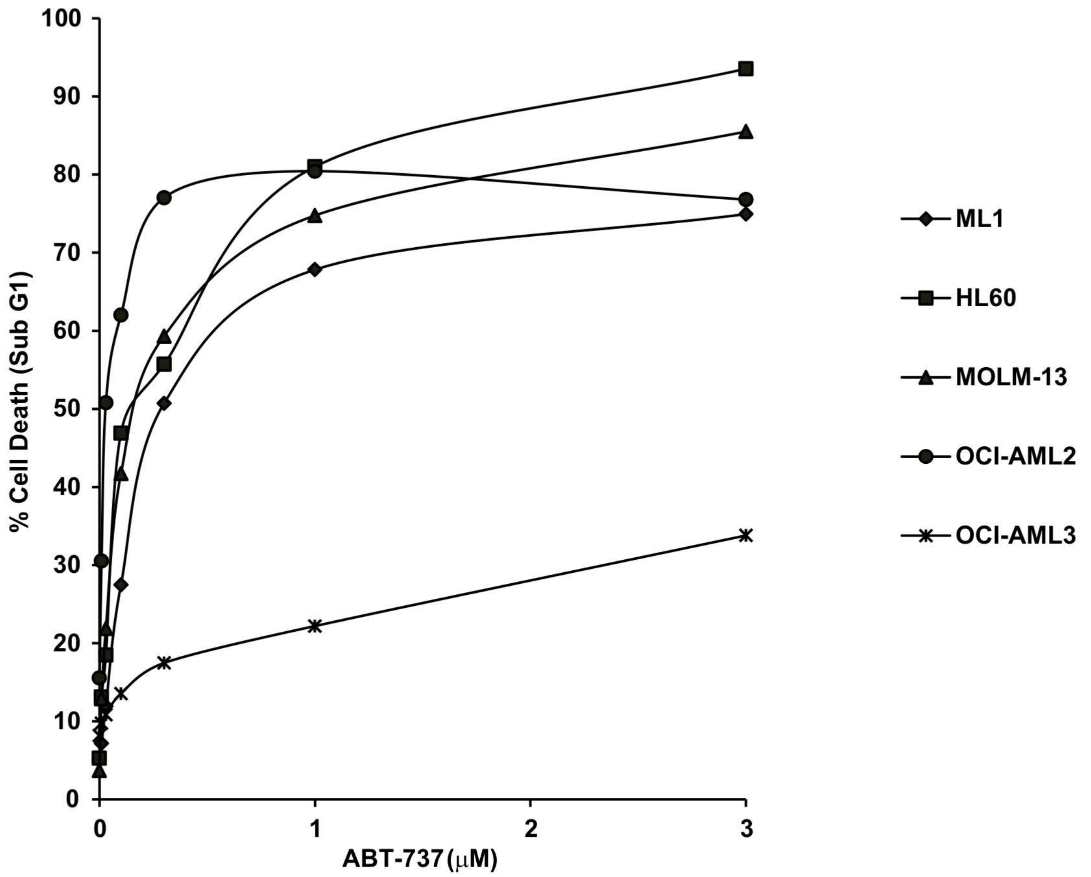

Supplementary Figure 3. ABT-737 dose response of AML cell lines. AML cell lines (ML-1, HL60, Molm-13, OCI-AML2 and OCI-AML-3) were treated with the indicated doses of ABT-737 for 24 h and induction of cell death was quantified by determining the sub-G1 population using propidium iodide staining and flow cytometry.

Supplementary Figure 4

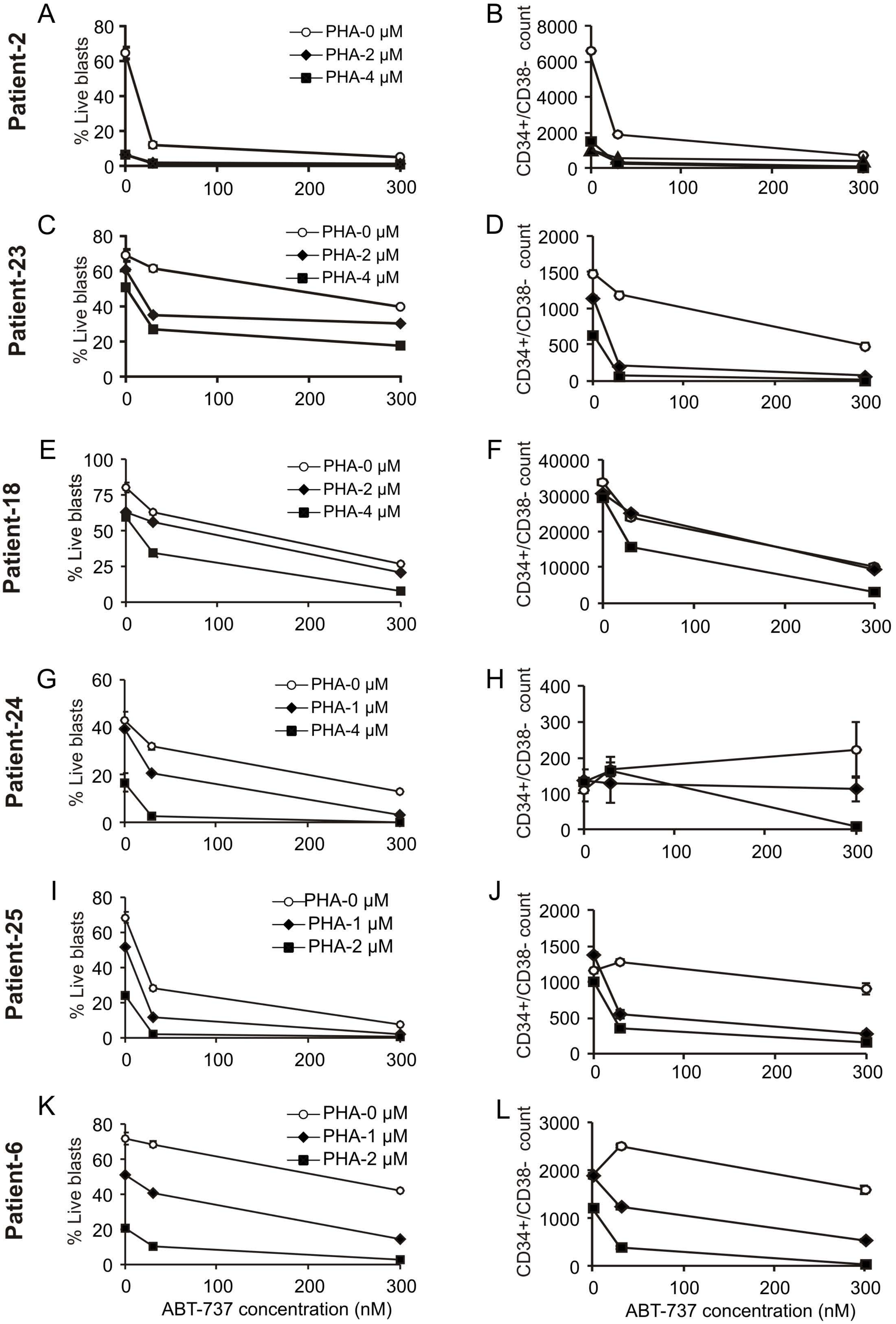

Supplementary Figure 4 ctd.

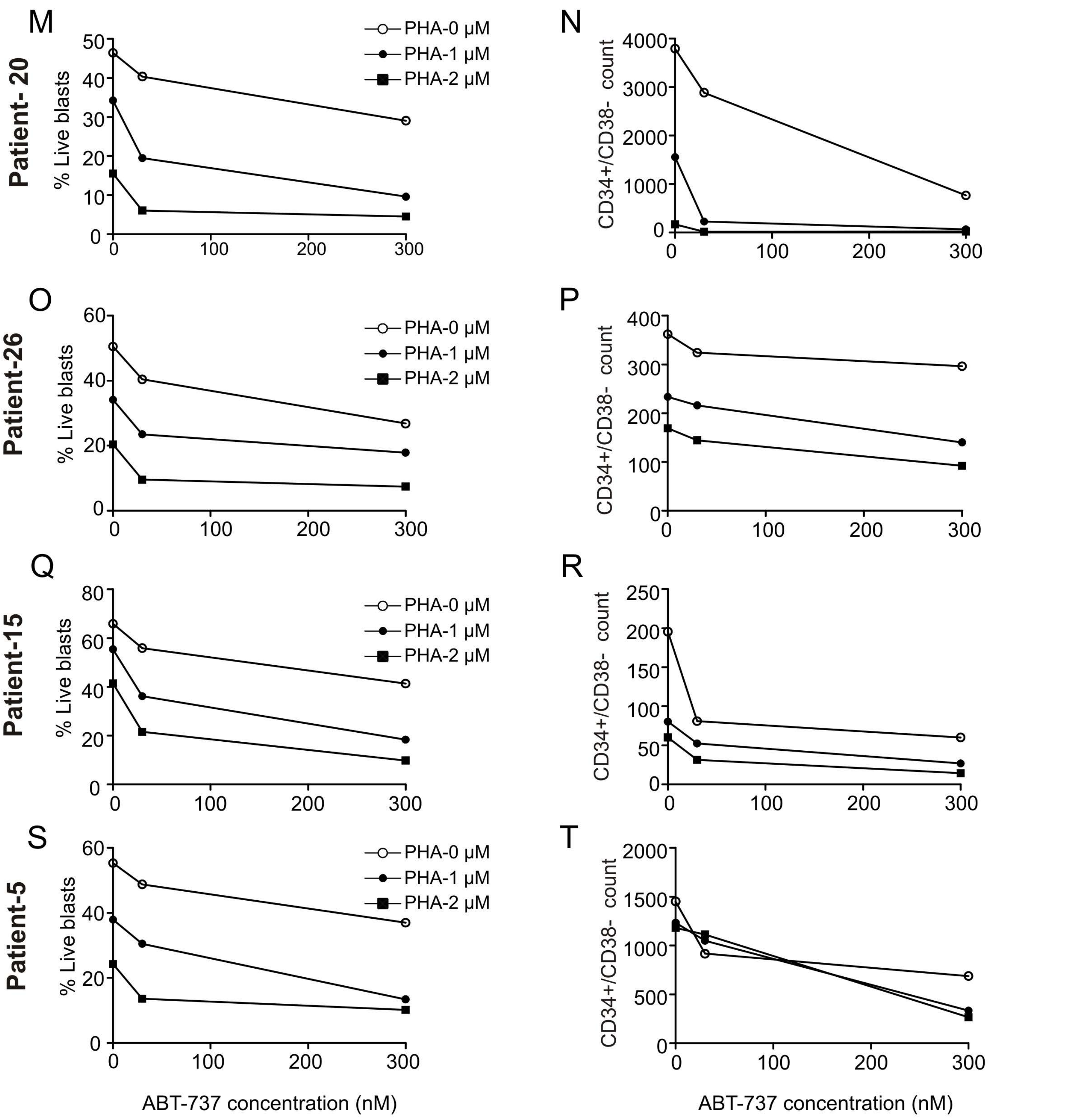

Supplementary Figure 4. Inhibition of Mcl-1 expression with PHA-767491 reverts BMSC-driven drug resistance of both AML blasts and CD34+/CD38- population. AML blasts from 10 patients were cultured on HS5-BMSC monolayer for 24 hours and then treated with ABT-737 (30 and 300 nM) with or without a 4 h pre-treatment with the CDC7/CDK9 inhibitor, PHA-767491 (1-4 µM). Induction of cell death was quantified with ToPro-3 viability staining both in the bulk AML population and the CD34+/CD38- LSC-encompassing population. Graphs on the left-hand side (A, C, E, G, I, K, M, O, Q and S) show the percentage of live cells in the bulk blast population, while the graphs on the right-hand side (B, D, F, H, J, L,N, P, R and T) show the number of CD34+/CD38- cells within the surviving population. The effect of PHA-767491 alone is shown by the points on the Y axis (zero ABT-737 concentration) and the effect of ABT-737 alone is shown by the PHA 0 µM line (open circles).

Supplementary Figure 5

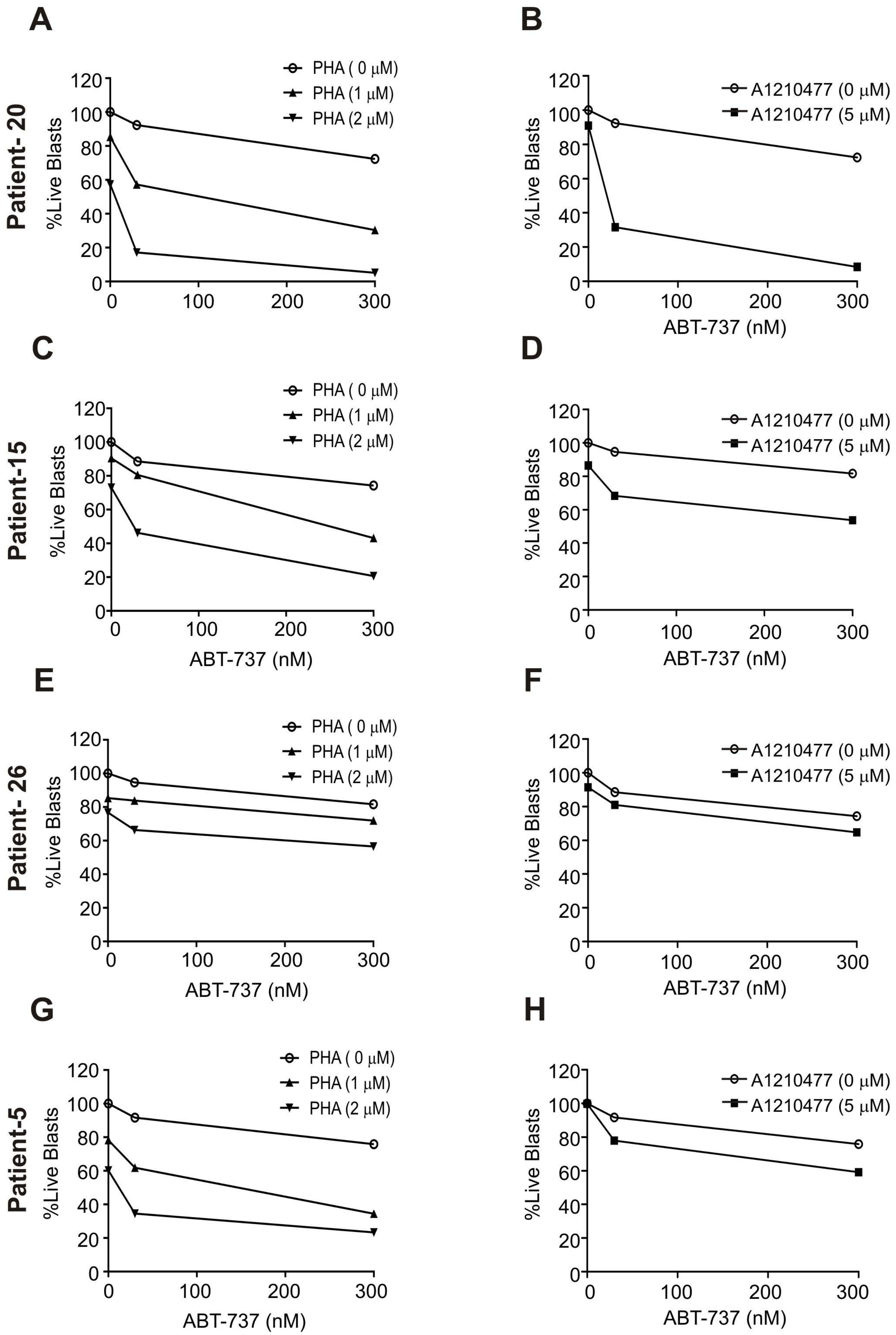

Supplementary Figure 5. Repression or inhibition of Mcl-1 sensitises AML blasts cultured on matched BMSCs to ABT-737. Viability of AML blasts in matched BMSC co-culture treated with ABT-737 plus PHA-767491 or A1210477. BM-derived MNCs from 4 AML patients were cultured with their matched BMSCs for 24 h. Cells were pre-treated with PHA-767491 (1-2 μM) (A, C, E, G) or A1210477 (B, D, F, H) for 4 h followed by ABT-737 (30 and 300 nM) for 24 h. Induction of cell death was measured by ToPro-3 viability staining and flow cytometry.

Supplementary Figure 6

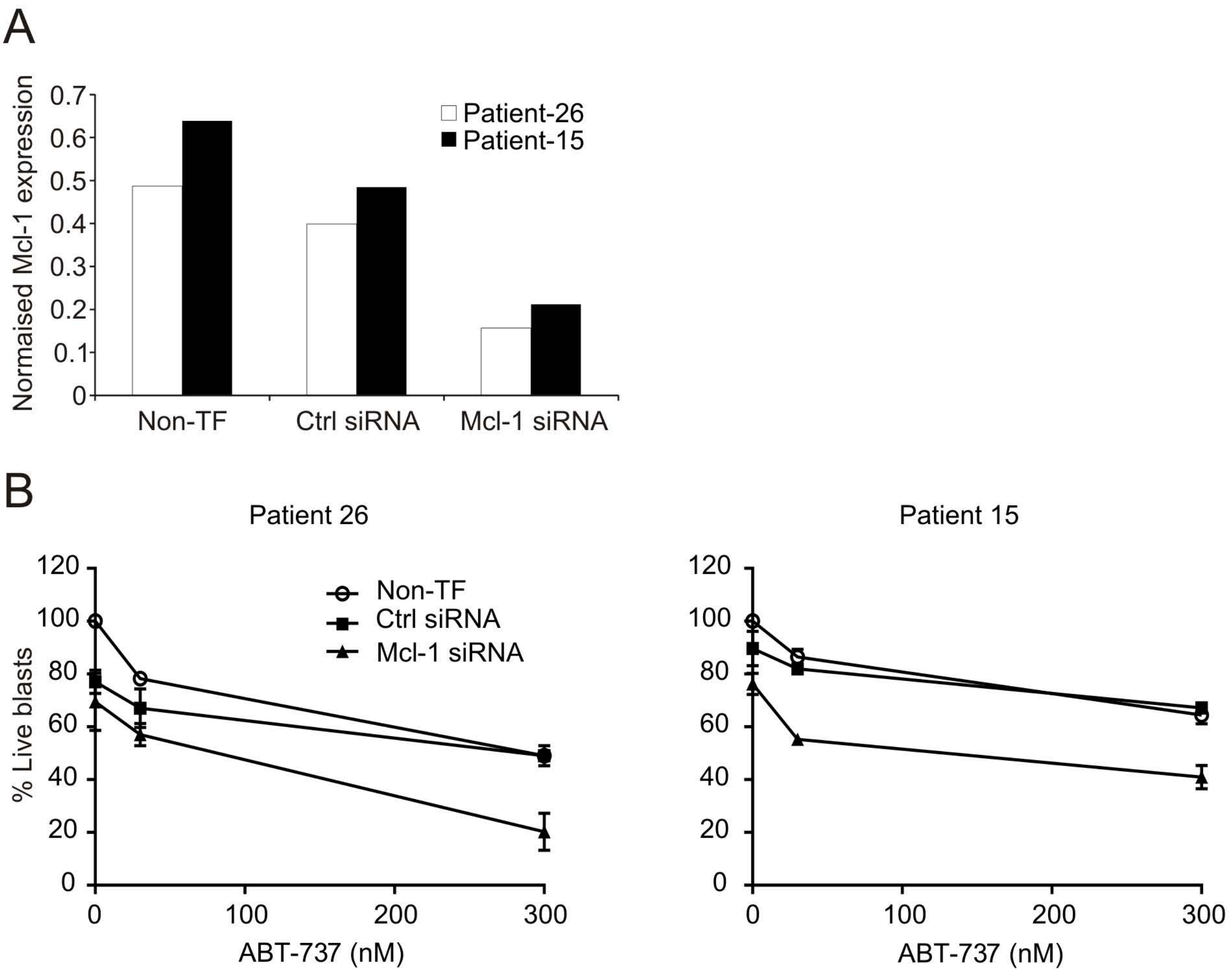

Supplementary Figure 6. Knockdown of Mcl-1 sensitises AML blasts to ABT-737. (A) Efficiency of siRNA-mediated Mcl-1 knockdown in primary AML blasts. Mcl-1 was transfected into primary AML blasts using nucleofection as described in Materials and Methods and Mcl-1 protein expression was quantified 48 h post-transfection using Western blotting. The graphs show densitometric quantification of Mcl-1 expression normalised to -actin. (B) Effect of Mcl-1 knockdown on ABT-737 sensitivity on AML blats. Mcl-1 was knocked down in two primary AML samples which showed resistance to ABT-737 and the AML blasts in co-culture with iMSC feeder layer were treated with ABT-737 for an additional 24 h. The graphs show induction of cell death determined with ToPro-3 staining and flow cytometry.

Supplementary Figure 7

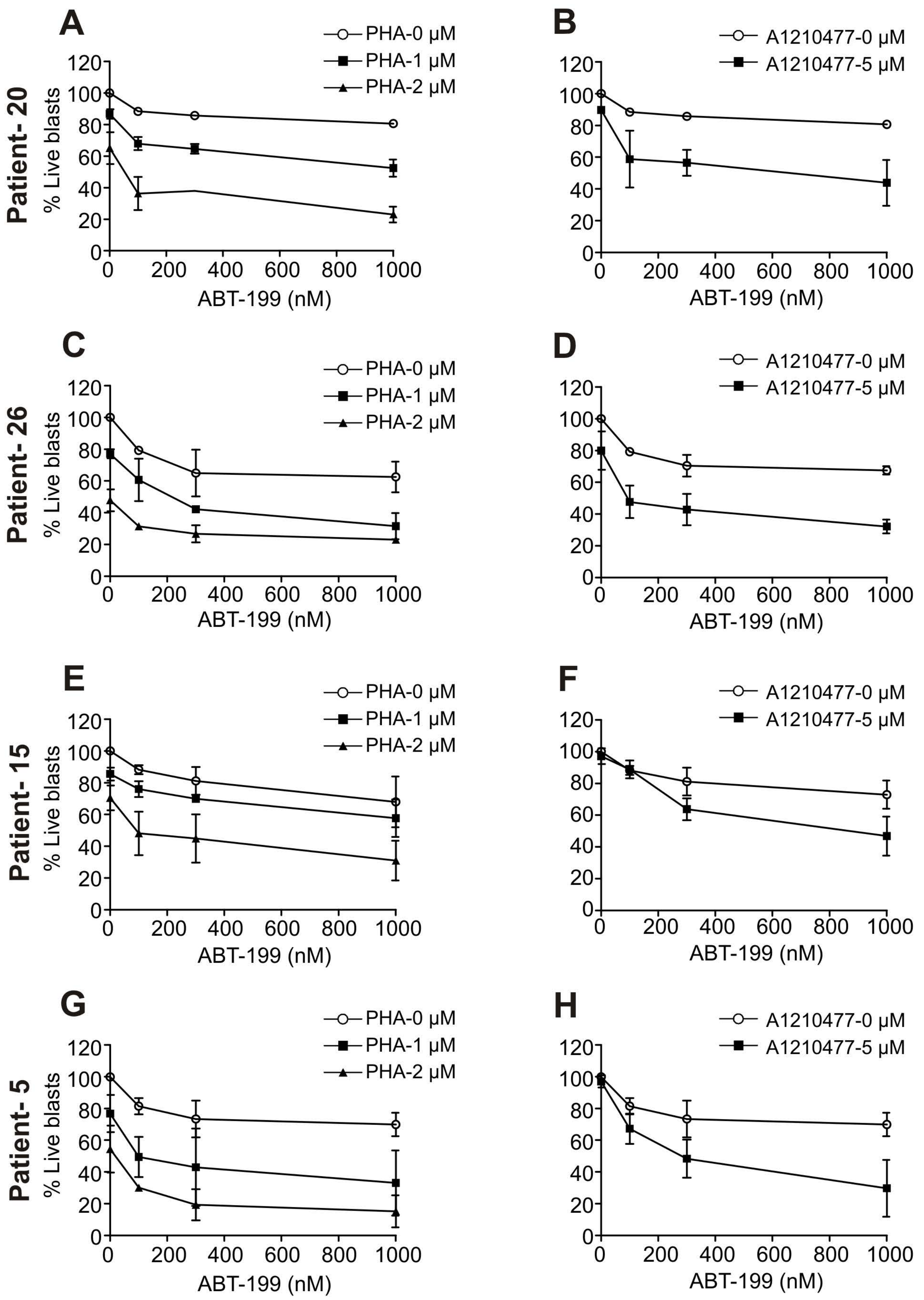

Supplementary Figure 7. Repression or inhibition of Mcl-1 sensitises AML blasts cultured in contact with BMSCs (iMSC) to ABT-199. Viability of AML blasts treated with ABT-199 plus PHA-767491 or A1210477. BM-derived MNCs from 4 AML patients were cultured with iMSC BMSCs for 24 h after which the cultures were pre-treated with the indicated doses of PHA-767491 (A, C, E, G) or A1210477 (B, D, F, H) for 4 h followed by ABT-737 for 24 h. Induction of cell death was measured by ToPro-3 viability staining. The graphs show percentage viability normalised to the untreated control.

Supplementary Figure 8

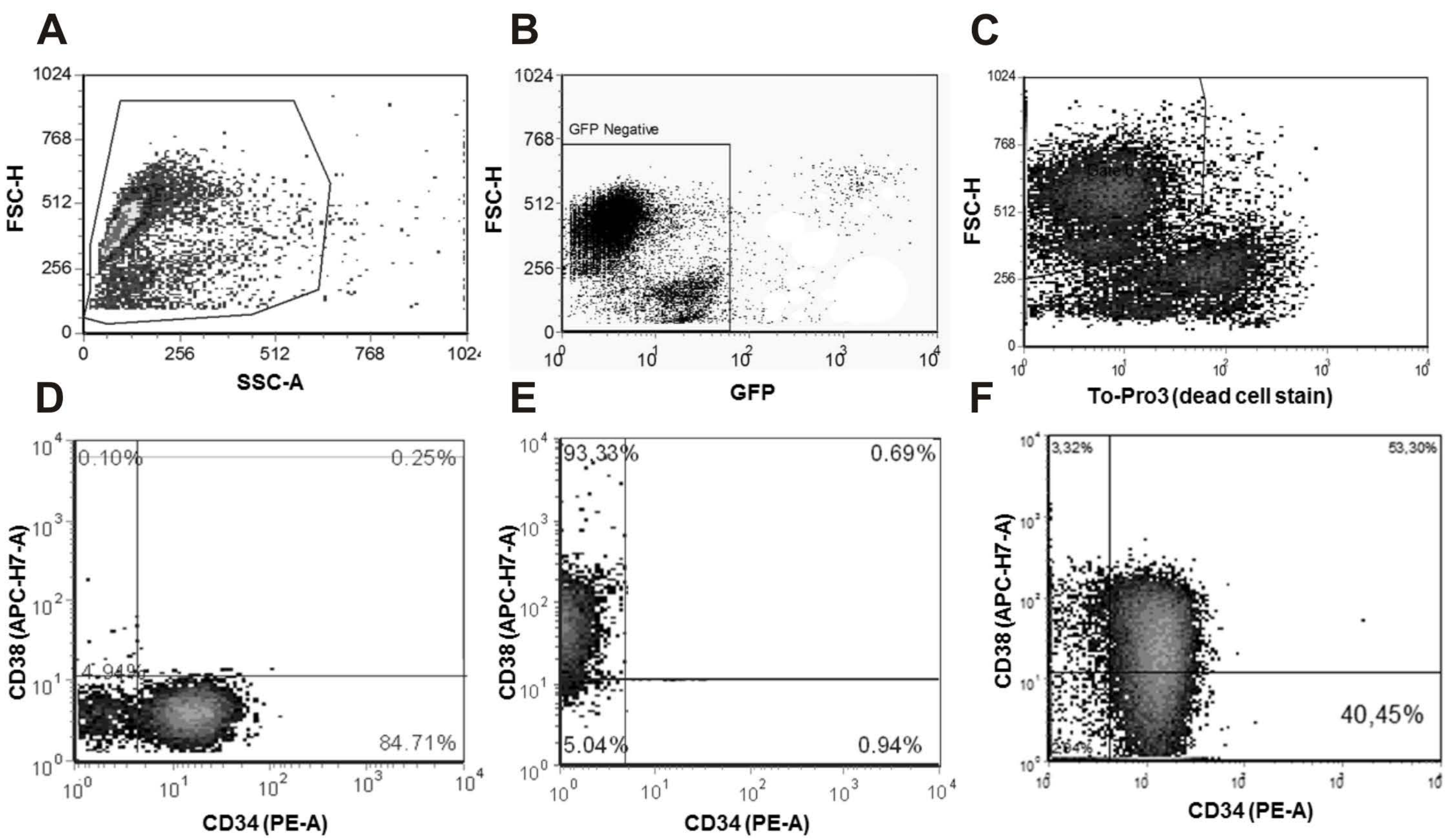

Supplementary Figure 8. Flow cytometry gating strategy. (A) Forward scatter (FSC) vs. side scatter (SSC) plot showing all events and gating of all cells. (B) FSC vs. GFP fluorescence dot plot and the gating to exclude detached GFP-positive HS-5 cells or CFSE-loaded iMSCs and primary BMSCs. (C) FSC vs. ToPro-3 fluorescence intensity dot plot used for determining the percentage of dead AML blasts (in the bulk blasts population). (D) CD38 (APC-H7) vs. CD34 (PE) dot plot in a sample stained only with anti-CD34 antibody to determine the CD34 gate. (E) CD38 (APC-H7) vs. CD34 (PE) dot plot in a sample stained only with anti-CD38 antibody to determine the CD38 gate. (F) CD38 (APC-H7) vs. CD34 (PE) fluorescence intensity dot plot showing the gating strategy for the $CD34^{+}$ and $CD38^{-}$ population.

Supplementary Table 1. Cytokines and chemokines secreted by HS-5 BMSCs.

Cytokines	Gene ID
**Angiogenin**	**283**
**Angiopoietin-1**	**284**
**Angiopoietin-2**	**285**
**BDNF**	**627**
CD30/TNFRSF8	943
**CD40L**	**959**
**YKL-40/CHITINASE-3 LIKE 1**	**1116**
**Dkk1/Dickkopf-1**	**22943**
**CD26/Dipeptidyl-peptidase IV**	**1803**
**CD147/EMMPRIN**	**682**
**Endoglin/CD105**	**2022**
**FGFbasic**	**2247**
FGF-19	9965
**G-CSF**	**1440**
**GDF-15**	**9518**
**GM-CSF**	**1437**
**ICAM-1**	**3383**
**IFNγ**	**3458**
**IL-1α**	**3552**
IL-1β	3553
**IL-6**	**3569**
**IL-11**	**3589**
**IL-17A**	**3605**
KLK3/Kallikrein-3	354

Cytokines ctnd	Gene ID
**LIF**	**3976**
**M-CSF**	**1435**
MIF	4282
**MMP-9/Gelatinase B**	**4318**
**Osteopontin**	**6696**
**PDGF-AA**	**5154**
**Pentraxin-3**	**5806**
**Serpin E1**	**5054**
**Thrombospondin-1**	**7057**
**PLAUR**	**5329**
**VEGF**	**7422**
**Vitamin D BP**	**2683**
**Chemokines**	**Gene ID**
**CXCL5/ENA-78**	**6374**
**CXCL1/GRO-α**	**2919**
**CXCL10/IP-10**	**3627**
**CCL2/MCP-1**	**6347**
**MCP-3/CCL7**	**6354**
**CCL20/MIP3α**	**6364**
**RANTES/CCL5**	**6352**
**SDF1α/CXCL12**	**6387**
**IL-8/CXCL8**	**3576**

Note: cyto/chemokines with bold typing are factors known to be present in the BMM.

Supplementary Table 2. Chow-Talalay's Combination Indexes (CIs) for primary AML samples treated with the combination of PHA-767491 and ABT-737

Patient 2

	PHA-767491, 1.0µM	PHA-767491, 2.0µM	PHA-767491, 4.0µM
ABT-737, 30.0nM	0.01356	0.00819	0.00414
ABT-737, 300.0nM	0.06914	0.02862	0.01146

Patient 23

	PHA-767491, 1.0µM	PHA-767491, 2.0µM	PHA-767491, 4.0µM
ABT-737, 30.0nM	0.52014	0.10794	0.06534
ABT-737, 300.0nM	0.40607	0.51382	0.17972

Patient 18

	PHA-767491, 1.0µM	PHA-767491, 2.0µM	PHA-767491, 4.0µM
ABT-737, 30.0nM	2.04139	0.8538	0.18457
ABT-737, 300.0nM	0.91403	0.67958	0.17622

Patient 24

	PHA-767491, 1.0µM	PHA-767491, 2.0µM	PHA-767491, 4.0µM
ABT-737, 30.0nM	0.63256	0.28856	0.38192
ABT-737, 300.0nM	0.22168	0.21843	0.11431

Patient 25

	PHA-767491, 1.0µM	PHA-767491, 2.0µM	PHA-767491, 4.0µM
ABT-737, 30.0nM	0.53429	0.30939	0.44438
ABT-737, 300.0nM	0.27690	0.27372	0.35501

Patient 6

	PHA-767491, 1.0µM	PHA-767491, 2.0µM	PHA-767491, 4.0µM
ABT-737, 30.0nM	0.86787	0.51221	0.57917
ABT-737, 300.0nM	0.53954	0.24697	0.32821

Patient 20

	PHA-767491, 1.0µM	PHA-767491, 2.0µM	
ABT-737, 30.0nM	0.60271	0.61593	
ABT-737, 300.0nM	0.42895	0.5483	

Patient 26

	PHA-767491, 1.0µM	PHA-767491, 2.0µM	
ABT-737, 30.0nM	0.56897	0.76301	
ABT-737, 300.0nM	0.49271	0.7228	

Patient 15

	PHA-767491, 1.0μM	PHA-767491, 2.0μM	
ABT-737, 30.0nM	0.47218	0.49642	
ABT-737, 300.0nM	0.27142	0.27859	

Patient 5

	PHA-767491, 1.0μM	PHA-767491, 2.0μM	
ABT-737, 30.0nM	0.51069	0.7748	
ABT-737, 300.0nM	0.36243	0.57367	

Supplementary Table 3. Clinical data of patient samples

Sample	New diagnosis or relapsed	Age	AML subtype	Cytogenetics (normal vs complex karyotype (NK, CK), etc)	Response to treatment
1	New diagnosis	57	AML w/o maturation	NK, FLT3-ITD^{-}	Refractory
2	New diagnosis	71	RAEB2	NK, FLT3-ITD^{-}	Responder
3	New diagnosis	70	AML w. MDS-related changes	CK	Refractory
4	Relapsed	68	AML	NK, FLT3-ITD^{-}, NPM1^{+}	Refractory
5	New diagnosis	91	N/A	N/A	Supportive care
6	New diagnosis	64	AMML	NK, FLT3-ITD^{+}	Initial responder, then relapse
7	New diagnosis	68	RAEB2	NK, FLT3-ITD^{+}	Refractory
8	New diagnosis	53	AML	CK, FLT3-ITD^{-}	Refractory
9	New diagnosis	62	AML	NK, FLT3-ITD^{+}	Responder
10	New diagnosis	71	AML-M1	NK, FLT3-ITD^{-}	Refractory
11	New diagnosis	45	AML	t(8:21), good risk	Responder
12	New diagnosis	47	MDS	N/A	Responder
13	Relapsed	52	t-AML	NK	Refractory
14	New diagnosis	85	MDS	N/A	Refractory
15	New diagnosis	39	AML	NK, FLT3-ITD^{+}	Responder
16	New diagnosis	66	AML	CK, Poor risk	Refractory
17	New diagnosis	50	AML	NK, Intermediate risk	Responder
18	New diagnosis	73	AML-M1	N/A	Refractory
19	Relapsed	63	sAML	NK	Responder
20	New diagnosis	70	AML-M1	NK, FLT3-ITD^{-} ,NPM1^{-}	Responder
21	Relapsed	77	sAML	CK	Refractory
22	Relapsed	76	AML	CK, FLT3-ITD^{+}	Refractory
23	New diagnosis	73	AML	NK	Responder
24	New diagnosis	68	AML	NK, FLT3-ITD^{-} , NPM1^{+}	Refractory

25	New diagnosis	91	AML	N/A	Supportive care
26	New diagnosis	69	sAML	NK	Refractory
27	New diagnosis	N/A	Multiple myeloma	N/A	NA
28	New diagnosis	N/A	Hodgkins lymphoma	N/A	NA
29	New diagnosis	N/A	Multiple myeloma	N/A	NA
30	New diagnosis	N/A	Ewing's Sarcoma	N/A	NA

Abbreviations: **AML**, acute myeloid leukemia; **RAEB2**, refractory anemia with excess blasts-2; **MDS**, myelodysplastic syndromes; **AMML**, acute myelomonocytic leukemia; **FLT3**, Fms-like tyrosine kinase-3; **ITD**, internal tandem duplication; **NPM1**, nucleophosmin-1, **sAML,** secondary AML and **tAML,** therapy-related AML.
